# Effects of Harvest Timing on Selected Aroma-Related Volatile Compounds in Blaufränkisch and Pinot Noir Wines

**DOI:** 10.3390/foods15162800

**Published:** 2026-08-10

**Authors:** Nikolaus Schlögl, Sezer Sari, Phillip Eder, Reinhard Eder, Christian Philipp

**Affiliations:** Federal College and Research Institute for Viticulture and Pomology Klosterneuburg (HBLAuBA Klosterneuburg), 3400 Klosterneuburg, Austria; nikolaus.schloegl@weinobst.at (N.S.); sezer.sari@weinobst.at (S.S.); phillip.eder@weinobst.at (P.E.); reinhard.eder@weinobst.at (R.E.)

**Keywords:** polyfunctional thiols, C_13_-norisoprenoids, monoterpenes, rotundone, C_6_ alcohols, red wine aroma, grape ripening, harvest timing, Blaufränkisch, Pinot Noir

## Abstract

Harvest timing is an important management factor associated with the volatile composition of wine. This one-vintage study investigated selected free aroma-related volatile compounds in Blaufränkisch and Pinot Noir wines produced under standardised microvinification from grapes harvested at early, standard and late maturity-related stages in four commercial vineyards. Free volatile polyfunctional thiols, six-carbon (C_6_) alcohols, free monoterpenes, rotundone and free 13-carbon (C_13_) norisoprenoids were quantified using gas chromatographic methods, and data were evaluated using exploratory univariate and multivariate statistics. Harvest-stage responses were compound-specific and depended on the investigated grape-variety/regional sample sets. The concentration of 1-hexanol generally decreased with advancing harvest stage, whereas 3-sulfanylhexan-1-ol (3-SH) tended to increase across several vineyard origins. An exploratory pooled correlation between 1-hexanol and 3-SH was weakly negative, indicating only a limited association between the two compounds in bottled wines. Free monoterpenes and free C_13_-norisoprenoids showed non-linear, compound-dependent patterns, while rotundone showed no consistent overall harvest-stage response. Principal component analysis (PCA) separated the wines mainly according to grape-material background, with additional harvest-stage-related structuring. Overall, under the specific conditions of this one-vintage study, operationally defined maturity-related harvest timing was associated with compound- and background-dependent changes in selected free aroma-related volatile compounds in the investigated red wines.

## 1. Introduction

Harvest timing determines the maturity status and chemical composition of the grape material entering vinification and may consequently influence the aroma-related volatile composition of the resulting wine. Relevant compound classes include six-carbon (C_6_) alcohols, free volatile polyfunctional thiols, free monoterpenes, free 13-carbon (C_13_) norisoprenoids and rotundone; these classes may respond differently to berry ripening, precursor availability, and subsequent winemaking processes.

*Vitis vinifera* L. cvs. Blaufränkisch and Pinot Noir are commercially important red grape varieties in Central European wine production. Blaufränkisch, also known as Lemberger, Kékfrankos, or Frankovka modrá, is an established Central European red grape variety. Previous studies have characterised selected free monoterpenes and C_13_-norisoprenoids in Blaufränkisch grapes and wines [1,2]. Pinot Noir, also known as Blauer Burgunder, Blauburgunder, or Spätburgunder, is one of the most internationally recognised red grape varieties and is typically associated with a subtle aroma profile characterised by fruity, floral, and spicy nuances. In this variety, norisoprenoids, selected free monoterpenes, and other volatile compounds may contribute to the overall aroma composition [1,3]. These two cultivars were selected because they represent commercially relevant red grape materials in Central European wine production and because previous studies have characterised several of the targeted aroma-related compound classes in these cultivars [1,2,3]. Moreover, the investigated Blaufränkisch and Pinot Noir materials differed in their practical ripening and commercial harvest windows, thereby providing a suitable applied framework for examining whether harvest-stage-related responses were consistent across contrasting grape-material backgrounds. The selection was not intended to support a general comparison of the two cultivars, but to investigate maturity-related changes within commercially relevant grape materials under standardised vinification conditions.

The C_6_ alcohols are formed via the lipoxygenase pathway from polyunsaturated fatty acids and are commonly associated with green and herbaceous sensory notes in grapes and wines [4,5]. Major representatives include 1-hexanol and *cis*-3-hexen-1-ol, together with their corresponding aldehydes. These C_6_ compounds are often more abundant in less mature grapes and may decrease during ripening, which has been attributed to changes in lipoxygenase activity and precursor availability [5,6]. Because pronounced green notes may affect the perceived balance of red wines, understanding the evolution of C_6_ alcohols during grape ripening is relevant for aroma management and harvest decisions.

Free volatile polyfunctional thiols such as 3-sulfanylhexan-1-ol (3-SH) and 4-methyl-4-sulfanylpentan-2-one (4-MSP) are highly potent aroma compounds that contribute to fruity and varietal aroma impressions in wine [7,8]. In grapes, these compounds occur mainly as odourless cysteinylated or glutathionylated precursors, which can be converted into volatile thiols during alcoholic fermentation through yeast enzymatic activity [9,10]. The accumulation of thiol precursors during ripening is cultivar-dependent and may vary according to grape maturity [9,11,12,13]. Their biochemical formation is linked to C_6_ metabolism, as unsaturated C_6_ aldehydes and related metabolites can participate in the formation of thiol conjugates in grapes [14,15]. Therefore, the simultaneous determination of C_6_ alcohols and free volatile thiols in finished wines can characterise their maturity-related co-variation and final concentrations, but cannot directly resolve changes in the underlying cysteinylated or glutathionylated precursor pools.

Free monoterpenes such as linalool, geraniol, nerol, citronellol, and α-terpineol are mainly associated with floral and fruity aroma attributes in grapes and wines [3,16]. In grape berries, they occur both as free volatile compounds and as glycosidically bound precursors, which may be released during winemaking and storage. Their concentrations are strongly cultivar-dependent, with aromatic cultivars such as Muscat varieties typically showing high levels, whereas many red cultivars, including Blaufränkisch and Pinot Noir, generally contain lower concentrations [1,3,16]. During ripening, glycosidically bound monoterpenes often increase, whereas free monoterpenes may show more variable and compound-specific trends [17,18]. This highlights the need to evaluate individual compounds rather than treating monoterpenes as a uniform chemical group.

The C_13_-norisoprenoids, including β-damascenone, β-ionone, vitispirane, and 1,1,6-trimethyl-1,2-dihydronaphthalene (TDN), originate mainly from carotenoid degradation and can contribute to floral, fruity, honey-like, spicy, or aged aroma nuances even at low concentrations [19,20,21]. Their formation is closely related to grape carotenoid metabolism and the availability of glycosidically bound precursors. Several studies have shown that norisoprenoid concentrations or precursor pools may increase during ripening, although individual compounds can also exhibit non-linear trends, especially at advanced maturity stages or during wine maturation [2,3,22,23]. For this reason, free C_13_-norisoprenoids are relevant when evaluating the relationship between harvest timing and the aroma-related volatile composition of red wines.

Rotundone is a sesquiterpene that contributes to characteristic peppery aroma notes in wine and has been detected in several red and white grape varieties [24]. High concentrations have been reported in cultivars such as Syrah, Grüner Veltliner, Duras, and Mourvèdre, whereas Pinot Noir typically shows lower concentrations that may be close to the sensory threshold [25]. The compound is mainly localised in grape skins and has often been reported to accumulate after véraison, particularly during later stages of berry ripening [24,26,27,28]. However, the response of rotundone to harvest timing may differ from that of other grape-derived aroma compounds and therefore warrants separate evaluation.

Despite the recognised influence of grape maturity on aroma development, limited information is available on how operationally relevant harvest timing affects several selected aroma-related volatile compound classes simultaneously in finished red wines. Previous studies have frequently focused on individual compound classes, grape precursor pools, or single cultivars, whereas integrated investigations of C_6_ alcohols, free volatile polyfunctional thiols, free monoterpenes, free C_13_-norisoprenoids, and rotundone across early, standard, and delayed harvest stages under standardised vinification conditions remain scarce. This knowledge gap is particularly relevant for Blaufränkisch and Pinot Noir, for which compound-specific ripening studies exist, but the coordinated expression of these volatile classes in wines produced from contrasting maturity-related harvest stages has not been sufficiently characterised.

Against this background, the present study aimed to investigate changes in selected free aroma-related volatile compounds in wines produced from commercially relevant Blaufränkisch and Pinot Noir grape materials harvested at three maturity-related harvest stages. Particular emphasis was placed on compound-class-specific responses of free volatile polyfunctional thiols, C_6_ alcohols, free monoterpenes, rotundone, and free C_13_-norisoprenoids to harvest timing under standardised microvinification conditions. Because grape variety, wine-growing region, and vineyard origin were not fully crossed in the experimental design, differences between the two materials were interpreted as differences between the investigated grape-variety/regional sample sets rather than as general varietal effects. It was hypothesised that maturity-related harvest timing would be associated with compound-specific changes in selected aroma-related volatile compounds, with C_6_ alcohols decreasing during ripening and selected free volatile polyfunctional thiols, free monoterpenes, and free C_13_-norisoprenoids showing increasing or non-linear trends. Multivariate analysis was used to explore whether the overall volatile profiles reflected differences associated with harvest stage and the investigated grape materials.

## 2. Materials and Methods

### 2.1. Experimental Vineyard Material

The grape material used in this study originated from four commercial vineyards located in two Austrian wine-growing regions. Blaufränkisch grapes were obtained from two vineyards in Mittelburgenland, whereas Pinot Noir grapes were obtained from two vineyards in Thermenregion. The vineyard origins were selected to provide commercially relevant grape materials for each investigated cultivar and to permit replicated evaluation of harvest-stage responses within two independent vineyard origins per grape-variety/regional sample set. However, the study was not designed as a direct comparison of vineyard sites, wine-growing regions, or grape varieties as independent factors. Throughout the manuscript, “vineyard origin” refers to each of the four individual commercial vineyards, whereas “grape-variety/regional sample set” refers to the grouped Blaufränkisch/Mittelburgenland and Pinot Noir/Thermenregion materials used in the exploratory two-way analyses. The more general term “grape material” is used when referring to the physical grapes or wines from a particular experimental origin.

Grape variety, wine-growing region, and vineyard-specific characteristics were not fully disentangled in the experimental design. The four vineyard origins differed in several descriptive characteristics, including year of planting, clone, rootstock, and elevation. These factors were not experimentally controlled and were partly nested within the investigated grape-variety/regional sample sets. Accordingly, differences between Blaufränkisch and Pinot Noir are interpreted throughout this study as differences between the investigated grape-variety/regional sample sets under the specific conditions of the 2023 vintage, rather than as general varietal effects independent of vineyard or regional background.

The study was, therefore, designed to evaluate maturity-related harvest-stage responses within commercially relevant grape materials, rather than to isolate the independent effects of grape variety, vineyard site, vine age, clone, rootstock, or wine-growing region. Detailed descriptive information on the vineyard material is provided in Table 1.

Vineyard management practices, including pruning, shoot positioning, leaf removal, and under-vine management, followed standard commercial practices at the respective sites. No cluster thinning was performed. Plant protection was carried out according to integrated pest management guidelines.

Daily gridded meteorological data were obtained from the SPARTACUS-v3 dataset of GeoSphere Austria at a spatial resolution of 1 km using the coordinates of the four vineyard origins [29]. The grid points returned by the data service were located between 0.22 and 0.64 km from the corresponding vineyard coordinates. The extracted variables comprised daily minimum air temperature (TN), daily maximum air temperature (TX), 24-h mean air temperature (TM24), and daily precipitation sum (RR). Climatic conditions were summarised for the period from 1 April 2023 to the respective late harvest date and separately for the experimental harvest window from EHD to LHD. Growing degree days above a base temperature of 10 °C (GDD_10_) were calculated from 1 April to the respective LHD as the sum of max(TM24 − 10 °C, 0). The Huglin heliothermal index was calculated for the standard Northern Hemisphere period from 1 April to 30 September using TM24, TX and a latitude-dependent day-length coefficient of 1.05 for all four vineyard origins; negative daily contributions were set to zero [30]. The resulting climatic indicators are summarised in Table 2.

Across the four vineyard origins, GDD_10_ accumulated from 1 April to the respective LHD ranged from 1437 to 1632 °C d, while the Huglin Index ranged from 1973 to 2132 °C d. Cumulative precipitation from 1 April to LHD ranged from 401.3 to 537.0 mm. During the respective experimental harvest windows from EHD to LHD, mean air temperature ranged from 17.6 to 19.1 °C, mean daily minimum temperature from 11.8 to 12.8 °C, and mean daily maximum temperature from 24.3 to 26.4 °C. Precipitation during these periods ranged from 15.2 to 24.3 mm. Because the Pinot Noir and Blaufränkisch harvest schedules differed by one week, these climatic data are presented as descriptive background conditions and not as independent effects of grape variety, vineyard origin, or wine-growing region.

### 2.2. Harvest, Grape Processing, and Vinification

The harvest dates corresponding to the three calendar-defined, maturity-related harvest stages are summarised in Table 3. The standard harvest date (SHD) was defined as the expected commercial harvest date for the respective vineyard and variety in the 2023 vintage. It was set prospectively before EHD based on expert assessment of the cultivar-specific ripening course and the anticipated commercial harvest window and subsequently coincided with the actual harvest date selected by the respective winery within ±1 day. No fixed numerical threshold for °Brix, pH, or titratable acidity was imposed, because SHD was intended to represent the practical harvest decision of the respective winery rather than a standardised physiological maturity endpoint. The one-week offset between the Pinot Noir and Blaufränkisch harvest schedules reflected the earlier commercial harvest window of the investigated Pinot Noir vineyards under the conditions of the 2023 vintage. Grapes assigned to the early harvest date (EHD) and late harvest date (LHD) were harvested 14 days before and 14 days after the respective SHD, respectively.

The 14-day interval was selected a priori as a practically relevant window that was sufficiently wide to capture ripening-related changes while remaining representative of realistic commercial decisions around harvest. The selection was also informed by a closely related previous ripening study in Blaufränkisch, in which two-week sampling intervals revealed marked changes in carotenoids and C_13_-norisoprenoids [31]. Harvest stage was therefore defined operationally by calendar date relative to the respective SHD and should not be interpreted as a set of fully independent physiological maturity classes. The corresponding must parameters, including °Brix, pH, and titratable acidity, were used to document the maturity status of the grape material at processing and are reported in Table 4. Accordingly, the three stages were intended to represent realistic practical harvest decisions—two weeks before, at, and two weeks after the expected commercial harvest date—rather than experimentally standardised physiological maturity classes. Throughout the manuscript, “harvest stage” refers to the categorical experimental factor comprising EHD, SHD, and LHD, “harvest date” refers to the corresponding calendar date, and “harvest timing” denotes the broader practical decision regarding when grapes are harvested.

Blaufränkisch and Pinot Noir grapes were harvested manually in white stacking crates (Raiffeisen Ware Austria, Guntramsdorf, Austria). For each vineyard origin × harvest stage combination, three independently harvested replicate grape lots were obtained from spatially distinct, predefined vine sets and were collected, transported, processed, and vinified separately. Each replicate lot consisted of approximately 10 kg of grapes, resulting in approximately 30 kg of grape material per vineyard origin × harvest stage combination. The three replicate grape lots were treated as biological replicates at the level of grape sampling and microvinification. This design was intended to capture within-origin variability in the harvested grape material while maintaining independent processing and fermentation for each replicate lot.

Vine selection followed predefined exclusion criteria to ensure representative sampling and to reduce systematic bias. Vines showing visible symptoms of ESCA or other grapevine diseases, such as Bois noir, were excluded. Varietal identity and phenological representativeness of the selected grape clusters were verified prior to harvest. In addition, the first ten metres of each row and the two outermost vineyard rows were excluded from sampling to minimise edge effects.

The harvested grapes showed no visible symptoms of major grapevine diseases, including powdery mildew (*Erysiphe necator*), downy mildew (*Plasmopara viticola*), or *Botrytis cinerea*. Immediately after harvest, each replicate grape lot was destemmed and crushed separately at the Department of Oenology of the HBLAuBA for Viticulture and Pomology in Klosterneuburg, Austria. Processing was carried out using a small-scale destemming and crushing device suitable for microvinifications, ensuring separate handling of all replicate lots and treatments.

Basic must parameters were determined for all treatments by Fourier-transform infrared spectroscopy (FT-IR) according to OIV/OENO Resolution 390/2010 [32] and are summarised in Table 4. Unless stated otherwise, must parameters refer to the musts before chaptalisation. For SHD and LHD, must parameters were determined separately for the three replicate grape lots. For EHD, must parameters were determined from one pooled must sample prepared from the three replicate grape lots; therefore, no standard deviation is reported for EHD must parameters. These EHD must data were used only to document the maturity status of the grape material at processing. Aroma analyses and wine parameters, however, were performed on the independently vinified biological replicates for all harvest stages.

During processing, mash temperatures were maintained below 15 °C. For microbial stabilisation, potassium metabisulfite (K_2_S_2_O_5_; Lallemand, Guntramsdorf, Austria) was added to the mash at a rate of 10 g/100 kg. The mashes were subsequently transferred separately into 10-L fermentation vessels. Three independently harvested and vinified biological replicates were prepared for each vineyard origin × harvest stage combination, resulting in 18 fermentations for Blaufränkisch and 18 fermentations for Pinot Noir. The mash mass per fermentation vessel was 6.5 kg ± 0.5 kg.

The commercial yeast strain LA Bayanus (*Saccharomyces cerevisiae*; Lamothe-Abiet, Canéjan, France) was added after rehydration at a dosage of 60 g/100 kg mash. Malolactic fermentation was not intended; therefore, no co-inoculation with lactic acid bacteria was performed. After the onset of alcoholic fermentation, sucrose was added where required to reduce differences in fermentable sugar concentration resulting from differences in initial grape maturity. Addition rates were calculated from the pre-chaptalisation must parameters using a target value of 25.0 °Brix. No sucrose was added to the GUN-LHD fermentations because the corresponding musts already exceeded the target before chaptalisation (25.3 ± 0.1 °Brix). This standardisation step was intended to improve comparability among treatments with respect to potential alcohol formation. However, it did not result in identical fermentable sugar concentrations or final alcohol concentrations among all treatments. These remaining differences were considered part of the treatment-related wine matrix and were taken into account when interpreting aroma-related volatile concentrations.

Alcoholic fermentation was conducted at 24 ± 1 °C. Fermentation progress was monitored daily by density measurements using a portable oscillating U-tube density meter (DMA 35, Anton Paar, Graz, Austria) and by visual inspection of fermentation activity. The cap was punched down manually twice per day. Additional nitrogen supplementation using diammonium hydrogen phosphate (DAHP) was not applied, as the measured yeast-assimilable nitrogen (YAN) concentrations were above 200 mg/L in all documented must samples and were therefore considered sufficient for the standardised microvinification conditions used in this study.

After seven days of maceration, the individual fermentations were pressed separately, and the resulting young wines were handled individually throughout racking, storage, and bottling. Basic analytical parameters of the resulting young wines are summarised in Table 5. All wines completed alcoholic fermentation, as indicated by non-quantifiable glucose and fructose concentrations. No evidence of malolactic fermentation was observed under the analytical conditions used, as lactic acid was non-detectable or below the limit of quantification in the resulting wines.

The first racking from the lees was performed on 5 November 2023 for all wines. The wines were subsequently transferred into 3-L glass demijohns and treated with potassium metabisulfite (K_2_S_2_O_5_) at a dosage of 16 g/100 L, corresponding to a nominal SO_2_ addition of approximately 90 mg/L. Final bottling of the experimental wines took place on 16 December 2023. Prior to bottling, the free SO_2_ concentration was adjusted to 60 mg/L. The wines were bottled in olive-green 500-mL glass bottles, type “BD Exklusiv” (Vetropack, Bülach, Switzerland), and sealed with tin–Saran screw caps (BT-Watzke GmbH, Pinggau, Austria). Bottles were stored at 16 °C ± 1 °C in the wine cellar of the Department of Oenology of the HBLAuBA Klosterneuburg until further analyses were conducted.

### 2.3. Analytical Design and Instrumentation

#### 2.3.1. Basic Must and Wine Parameters

Basic physicochemical parameters of the musts and wines were quantified at the Department of Chemistry of the Federal College and Research Institute for Viticulture and Pomology, Klosterneuburg, Austria. Analyses were performed using a FOSS WineScan FT 120 system (Foss, Hamburg, Germany) according to OIV/OENO Resolution 390/2010 [32]. Unless stated otherwise, must parameters were determined before chaptalisation.

For musts, the following parameters were determined: °Brix, density, glucose, fructose, titratable acidity, pH, tartaric acid, malic acid, volatile acidity, α-amino nitrogen expressed as o-phthaldialdehyde/N-acetyl-L-cysteine assay equivalent nitrogen (NOPA nitrogen), and ammonium. For wines, density, alcohol, glucose, fructose, titratable acidity, pH, tartaric acid, malic acid, lactic acid, volatile acidity, and citric acid were determined.

Yeast-assimilable nitrogen (YAN) was calculated from NOPA nitrogen and ammonium according to Bell and Henschke [33] using the following equation:

YAN = NOPA + ammonium × 0.8225

where YAN, NOPA, nitrogen, and ammonium are expressed as mg/L. The conversion factor 0.8225 accounts for the nitrogen fraction of ammonium when ammonium is expressed on a mass-concentration basis.

#### 2.3.2. Analysis of Aroma-Related Volatile Compounds

The quantification of free volatile polyfunctional thiols, C_6_ alcohols, free monoterpenes, free C_13_-norisoprenoids, and rotundone was carried out at the Department of Chemical Research and Isotope Analysis of the Federal College and Research Institute for Viticulture and Pomology, Klosterneuburg, Austria. Aroma analyses were performed approximately six months after bottling, following storage under the conditions described above. Thus, the reported concentrations represent the volatile composition of the bottled experimental wines after defined bottle storage and not the immediate post-fermentation volatile composition. Bottled wines from each biological replicate were analysed separately.

Because the target compounds differed substantially in polarity, volatility, and concentration range, separate gas chromatographic methods were used for the individual compound classes. These methods differed in sample preparation, chromatographic separation, detection mode, and internal standardisation. Compound-specific analytical performance characteristics, including calibration range, linearity, limits of detection and quantification, repeatability or precision, and recovery where available, are provided in Appendix A, Table A1. Where published methods were adapted to the present wine matrix or extended to additional analytes, the relevant adaptations and in-house validation data are reported in Appendix A, Table A1.

The C_6_ alcohols were quantified using an in-house HS-SPME-GC-MS method validated for the present wine matrix. The analytical workflow was based on established HS-SPME-GC-MS aroma-compound methods used at the institute. Analyses were performed on an Agilent 6890N gas chromatograph (Agilent Technologies, Santa Clara, CA, USA) coupled to a mass-selective detector and equipped with an HI-ACID (FFAP) capillary column (60 m × 0.25 mm i.d., 0.25 µm film thickness; Avantor, Milan, Italy). The quantified C_6_ alcohols were 1-hexanol, *cis*-3-hexen-1-ol, and *trans*-2-hexen-1-ol. Compound-specific performance data are provided in Appendix A, Table A1.

Free monoterpenes were analysed using headspace solid-phase microextraction gas chromatography–selected ion monitoring mass spectrometry (HS-SPME-GC-SIM-MS) following Philipp et al. [34]. In brief, wine samples were analysed using HS-SPME with a 65 µm PDMS/DVB fused-silica fibre (Supelco, Bellefonte, PA, USA), sodium chloride (Thermo Fisher Scientific, Waltham, MA, USA), and 3,4-dimethylanisole (Sigma-Aldrich, St. Louis, MO, USA) as internal standard. Analyses were performed on an Agilent GC-MS system (Agilent Technologies, Santa Clara, CA, USA) equipped with a TR-5MS capillary column (60 m × 0.25 mm i.d., 0.25 µm film thickness; Thermo Fisher Scientific, Waltham, MA, USA). Quantification was carried out using compound-specific target and qualifier ions. The quantified free monoterpenes were *trans*-linalool oxide, *cis*-linalool oxide, *trans*-limonene oxide, linalool, hotrienol, α-terpineol, β-terpineol, nerol oxide, nerol, citronellol, and geraniol.

The free C_13_-norisoprenoids β-damascenone, β-ionone, 1,1,6-trimethyl-1,2-dihydronaphthalene (TDN), and vitispirane were determined using an in-house adaptation of the automated HS-SPME-GC-MS/MS approach described by Philipp et al. [2]. In contrast to the grape-extract method described by Philipp et al. [2], the present analysis targeted free C_13_-norisoprenoids in bottled wines and was performed without acid hydrolysis. The analytical system consisted of a Trace 1300 gas chromatograph (Thermo Fisher Scientific, Waltham, MA, USA) coupled to a TSQ Duo triple quadrupole mass spectrometer (Thermo Fisher Scientific, Waltham, MA, USA) and equipped with a TriPlus RSH autosampler (Thermo Fisher Scientific, Waltham, MA, USA). Chromatographic separation was performed on an HI-ACID (FFAP) capillary column (60 m × 0.25 mm i.d., 0.25 µm film thickness; Avantor, Milan, Italy). Quantification was performed using compound-specific multiple reaction monitoring transitions and internal standardisation. Compound-specific validation data for the present wine samples are provided in Appendix A, Table A1.

Rotundone was quantified using a stable-isotope-dilution SPE-SPME-GC-MS/MS approach based on previously described methods for rotundone analysis in Austrian wines [35,36]. In brief, sample preparation involved solid-phase extraction followed by SPME prior to GC-MS/MS analysis in multiple reaction monitoring mode. The analytical system consisted of a Trace 1300 gas chromatograph coupled to a TSQ Duo triple quadrupole mass spectrometer (Thermo Fisher Scientific, Waltham, MA, USA) and equipped with a TriPlus RSH autosampler. Chromatographic separation was performed on a DB-5 capillary column (60 m × 0.25 mm i.d., 0.25 µm film thickness; Agilent Technologies, Santa Clara, CA, USA). Quantification was carried out using d_6_-rotundone as internal standard.

Free volatile polyfunctional thiols in the bottled wines were quantified using a method based on Sari et al. [37] that was subsequently adapted and further optimised for the present application. Only the unconjugated volatile compounds were determined; cysteinylated, glutathionylated, or other non-volatile thiol precursors in grapes, musts, or wines were not analysed. The method involved solid-phase extraction followed by derivatisation prior to GC-MS/MS analysis in multiple reaction monitoring mode. Chromatographic separation was performed on an HI-ACID (FFAP) capillary column (60 m × 0.25 mm i.d., 0.25 µm film thickness; Avantor, Milan, Italy). The quantified volatile thiols were 4-methyl-4-sulfanylpentan-2-one (4-MSP), 3-sulfanylhexan-1-ol (3-SH), and 3-sulfanylhexyl acetate (3-SHA). The LOD and LOQ values obtained from in-house validation of the adapted method are reported in Appendix A, Table A1; the remaining method–performance characteristics reported in Table A1 were taken from Sari et al. [37].

For all aroma analyses, calibration, internal standardisation, and compound identification were carried out according to the respective validated or adapted methods. Where compound-specific validation data were available in the cited literature, these were reported or referenced. Where published methods were adapted to the present matrix, extended to additional analytes or not fully documented for the specific compounds analysed here, the corresponding in-house performance data are provided in Appendix A, Table A1.

### 2.4. Statistical Analysis

Statistical analyses were performed using XLSTAT version 2023.3.1 (Lumivero, Denver, CO, USA). Prior to statistical analysis, concentration data were log10-transformed to improve normality and variance homogeneity. Only numerical concentration values above the respective compound-specific limit of quantification, or within the validated calibration range where formal LOQ values were not reported, were included in log-transformation and inferential statistical analyses of individual compounds. Values reported as not detected (n.d.) or detected but below the limit of quantification (n.q.) were treated as non-quantifiable and were not log-transformed or imputed. This approach avoided arbitrary substitution of left-censored values, but reduced the number of compounds and observations available for inferential analyses.

For descriptive sum parameters, including the sum of free monoterpenes and the sum of free C_13_-norisoprenoids, non-detected and non-quantifiable values were assigned a value of zero before summation on the original concentration scale. Thus, only quantifiable concentrations contributed positively to the respective sum parameters. This procedure was used only for calculating compound-class sums and was not applied to the statistical analysis of individual compounds. Sum parameters were interpreted cautiously because they combine compounds with different analytical behaviour, concentration ranges, and biochemical origins. Where sum parameters were included in inferential analyses, the calculated sums were log10-transformed in the same way as individual concentration data, provided that the resulting sum was greater than zero.

Compounds were included only in those inferential analyses for which quantifiable values were available across the factor levels required for the respective model. Compounds that were not detected or not quantifiable for a given model were excluded from that analysis and reported descriptively where appropriate. Where inferential analysis was not applicable because quantifiable values were not available across the required factor levels, results are marked as n.a. in the corresponding tables.

Normality was assessed using the Shapiro–Wilk test, while homogeneity of variances was evaluated using Levene’s test. Given the small number of biological replicates per vineyard origin × harvest stage combination, these assumption checks were interpreted as diagnostic rather than definitive tests. All inferential analyses were conducted on log10-transformed concentration data.

For exploratory screening of broad response patterns, two-way analysis of variance (ANOVA) was performed with calendar-defined, maturity-related harvest stage, investigated grape-variety/regional sample set, and their interaction as model terms. In this model, the factor “investigated grape-variety/regional sample set” refers to the two sample sets included in the study, namely Blaufränkisch from Mittelburgenland and Pinot Noir from Thermenregion. The two-way ANOVA was performed on data pooled across the respective vineyard origins within each sample set. To control the false discovery rate arising from multiple analytical endpoints, *p*-values were adjusted using the Benjamini–Hochberg procedure. For each of the sample set, harvest stage, and sample set × harvest stage interaction terms, adjustment was performed separately across the 18 endpoints for which the two-way ANOVA was applicable. Endpoints for which quantifiable values were not available across all factor levels required for the model were excluded from the respective correction families. The six endpoints excluded from the two-way ANOVA and the corresponding correction families were 3-SHA, *cis*-3-hexen-1-ol, *trans*-2-hexen-1-ol, *trans*-limonene oxide, β-terpineol, and TDN. Benjamini–Hochberg-adjusted *p*-values < 0.05 were considered indicative of statistically detectable differences. No post hoc test was applied to the two-way ANOVA results.

Because grape variety, wine-growing region and vineyard-specific characteristics were not fully crossed in the experimental design, the investigated grape-variety/regional sample set factor was not interpreted as a general grape-variety effect independent of vineyard or regional background. In addition, vineyard origin was not modelled as a separate independent factor because only two vineyard origins per sample set were available, which limited the suitability of nested or mixed-model approaches. Accordingly, the two-way ANOVA was used solely as an exploratory screening approach to identify broad patterns associated with harvest stage, sample set, and their interaction under the specific conditions of the study.

In addition to the exploratory two-way ANOVA, harvest-stage-related differences were assessed separately within each vineyard origin using one-way ANOVA, with harvest stage as the fixed factor. For these within-origin analyses, Tukey’s HSD post hoc test was used, where appropriate, to separate homogeneous groups. These within-origin analyses represented the primary inferential focus of the study because they describe harvest-stage responses within each grape material and avoid direct statistical comparisons between vineyard sites, wine-growing regions, or grape varieties. The *p*-values from the within-origin one-way ANOVAs were not subjected to an additional across-endpoint false-discovery-rate adjustment. Tukey’s HSD controlled the family-wise error rate for pairwise comparisons among the three harvest stages within each combination of analytical endpoint and vineyard origin.

An exploratory pooled Pearson product–moment correlation was calculated between log10-transformed 3-SH and 1-hexanol concentrations across all 36 independently harvested and vinified replicate wines. The test was two-sided. Because observations were pooled across sample sets, vineyard origins and harvest stages without modelling the grouped experimental structure, the correlation was interpreted only as a descriptive measure of overall co-variation and not as evidence of a causal relationship.

Principal component analysis (PCA) was performed as an exploratory multivariate tool to visualise patterns in the aroma-related volatile composition of the wines. The PCA was carried out in XLSTAT on the correlation matrix of log10-transformed concentration data. Only compounds with quantifiable values across all samples included in the PCA were used. Compounds not detected in any sample (3-SHA and *trans*-2-hexen-1-ol), as well as compounds with non-quantifiable values in one or more samples (*cis*-3-hexen-1-ol, *trans*-limonene oxide, β-terpineol, and TDN), were excluded from the PCA. The PCA was used to explore relationships among samples and compounds according to harvest stage and the investigated grape-variety/regional sample sets.

Because several individual compounds and calculated sum parameters were analysed, *p*-values from the exploratory two-way ANOVA were evaluated after Benjamini–Hochberg false-discovery-rate adjustment. The adjusted results should nevertheless be interpreted as exploratory and hypothesis-generating rather than confirmatory because of the non-fully crossed experimental design and the limited number of vineyard origins. The within-origin one-way ANOVAs and Tukey’s HSD comparisons were retained as the predefined primary analyses of harvest-stage responses within each grape material. Accordingly, emphasis was placed on the consistency of response patterns, effect direction, within-origin results, and compound-specific behaviour rather than on isolated *p*-values alone. Differences between the investigated sample sets were interpreted cautiously under the specific conditions of this study.

## 3. Results

### 3.1. Free Volatile Polyfunctional Thiols

The results for free volatile polyfunctional thiols are summarised in Table 6.

In the exploratory two-way ANOVA, 4-MSP concentrations differed between the investigated grape-variety/regional sample sets (Benjamini–Hochberg-adjusted *p* < 0.001) and showed harvest-stage-associated differences (adjusted *p* < 0.01). No statistically detectable sample set × harvest stage interaction was observed. Across the investigated wines, 4-MSP concentrations were generally lowest at EHD and increased towards SHD or LHD. Within-origin analyses indicated harvest-stage-related differences in LBG (*p* = 0.001), GUN (*p* < 0.001), and OBE (*p* = 0.001), where EHD showed lower concentrations than the later harvest stages. In contrast, no harvest-stage-related difference was detected in HOR.

For 3-SH, the exploratory two-way ANOVA showed harvest-stage-associated differences (Benjamini–Hochberg-adjusted *p* < 0.001), while no statistically detectable main difference between the investigated sample sets was observed. The sample set × harvest stage interaction was not statistically detectable after adjustment (adjusted *p* = 0.105). Overall, 3-SH concentrations tended to increase with advancing harvest stage. Within-origin analyses showed harvest-stage-related differences in all vineyard origins. In LBG, LHD showed higher 3-SH concentrations than EHD and SHD (*p* = 0.003), whereas EHD and SHD did not differ significantly. In HOR, GUN, and OBE, 3-SH increased progressively from EHD to SHD and LHD, with all harvest stages differing significantly from one another (*p* < 0.001).

A weak negative pooled Pearson correlation was observed between 3-SH and 1-hexanol across the investigated wines (r = −0.365; *p* = 0.028; R^2^ = 0.133). This indicates a weak inverse association between the concentrations of these two compounds in the present dataset. However, this relationship should be interpreted as exploratory and does not imply a direct causal relationship between the two compounds. The ester 3-SHA was not detected in any of the analysed treatments.

### 3.2. C_6_ Alcohols

The results for C_6_ alcohols are summarised in Table 7.

In the exploratory two-way ANOVA, 1-hexanol concentrations differed between the investigated sample sets and showed harvest-stage-associated differences, with Benjamini–Hochberg-adjusted *p*-values < 0.001 for both terms. A sample set × harvest stage interaction was also detected (adjusted *p* < 0.01), indicating that the harvest-stage response of 1-hexanol differed between the investigated grape materials. Overall, 1-hexanol concentrations tended to decrease from EHD towards SHD and LHD, although the magnitude of this decrease depended on vineyard origin and sample set.

This pattern was not uniform across all vineyard origins. In LBG, no harvest-stage-related difference in 1-hexanol concentrations was detected (*p* > 0.05), and concentrations remained approximately between 1.0 and 1.1 mg/L across all harvest stages. In HOR, 1-hexanol showed harvest-stage-related differences (*p* = 0.012), with higher concentrations at EHD than at SHD and LHD, while the two later harvest stages did not differ significantly from one another. In the Pinot Noir/Thermenregion sample set, a more pronounced harvest-stage-related decrease was observed in both vineyard origins (GUN and OBE; *p* < 0.001), where 1-hexanol concentrations were higher at EHD than at SHD and LHD.

For *cis*-3-hexen-1-ol, the exploratory two-way ANOVA was not applicable because quantifiable values were not available across all factor levels required for the model. Concentrations were low across the investigated wines and, where quantifiable, were generally close to 0.1 mg/L. Within-origin analyses showed no harvest-stage-related differences in HOR, GUN, or OBE (*p* > 0.05). In LBG, *cis*-3-hexen-1-ol was quantifiable only at LHD, whereas it was not detected at EHD and SHD; therefore, no within-origin inferential test was performed for this vineyard origin. The compound *trans*-2-hexen-1-ol was not detected in any of the analysed treatments.

### 3.3. Rotundone

The results for rotundone are summarised in Table 8.

In the exploratory two-way ANOVA, no statistically detectable differences were observed for the investigated grape-variety/regional sample set, harvest stage, or their interaction. Thus, no consistent overall harvest-stage-related response of rotundone concentrations was detected across the investigated wines.

Within-origin analyses, however, showed vineyard-origin-specific harvest-stage-related patterns. In the Blaufränkisch/Mittelburgenland sample set, no harvest-stage-related difference in rotundone concentrations was detected in LBG (*p* > 0.05), whereas differences were observed in HOR (*p* = 0.005), with LHD showing higher concentrations than EHD. In the Pinot Noir/Thermenregion sample set, harvest-stage-related differences were observed in both GUN and OBE (*p* < 0.001). In GUN, concentrations increased towards LHD, with all harvest stages differing significantly from one another. In OBE, by contrast, the highest concentrations were observed at SHD, while EHD and LHD did not differ significantly.

Overall, rotundone showed harvest-stage-related variation within individual vineyard origins, but the direction and magnitude of these responses differed among the investigated grape materials. These origin-specific patterns did not translate into statistically detectable overall differences associated with grape-variety/regional sample set, harvest stage, or their interaction in the exploratory two-way ANOVA. Therefore, rotundone did not show a uniform maturity-related response under the conditions of this study.

### 3.4. Free Monoterpenes

The results for free monoterpenes are summarised in Table 9.

Free monoterpenes showed compound-specific and partly non-linear responses to harvest stage. In the exploratory two-way ANOVA, several individual free monoterpenes showed differences associated with the investigated grape-variety/regional sample set and/or harvest stage. However, no uniform increase or decrease across the compound class was detected.

For *trans*-linalool oxide, the exploratory two-way ANOVA indicated differences associated with the investigated grape-variety/regional sample set (Benjamini–Hochberg-adjusted *p* < 0.001) and harvest stage (adjusted *p* < 0.05), while no statistically detectable sample set × harvest stage interaction was observed. However, within-origin analyses did not indicate harvest-stage-related differences in any of the four vineyard origins (*p* > 0.05). *Cis*-linalool oxide showed no statistically detectable differences associated with sample set, harvest stage, or their interaction in the exploratory two-way ANOVA. Within-origin analyses indicated harvest-stage-related differences only in HOR (*p* = 0.009), where concentrations were lower at SHD than at EHD and LHD.

For *trans*-limonene oxide, the exploratory two-way ANOVA was not applicable because quantifiable values were not available across all factor levels required for the model. Within the Blaufränkisch/Mittelburgenland sample set, harvest-stage-related differences were observed in both vineyard origins (LBG and HOR; *p* < 0.001). In both cases, concentrations were highest at SHD and lowest at LHD. In the Pinot Noir/Thermenregion sample set, *trans*-limonene oxide was not detected in GUN. In OBE, *trans*-limonene oxide was quantifiable at EHD and SHD, but not at LHD; therefore, the pattern was reported descriptively, and no within-origin inferential test was performed.

Linalool showed a difference associated with sample set (adjusted *p* < 0.001), whereas no statistically detectable main difference associated with harvest stage was observed (adjusted *p* = 0.097). However, a sample set × harvest stage interaction was detected (adjusted *p* < 0.05), indicating that the harvest-stage response differed between the investigated sample sets. Within-origin analyses showed decreasing concentrations from EHD towards later harvest stages in the Blaufränkisch/Mittelburgenland sample set, with harvest-stage-related differences in LBG (*p* = 0.001) and HOR (*p* = 0.020). In contrast, no harvest-stage-related differences were observed in the Pinot Noir/Thermenregion sample set.

Nerol oxide showed differences associated with sample set (adjusted *p* < 0.001) and harvest stage (adjusted *p* < 0.05), without a statistically detectable sample set × harvest stage interaction. Harvest-stage-related differences were observed in both Blaufränkisch vineyard origins, whereas no such differences were detected in the Pinot Noir vineyard origins.

Hotrienol differed between the investigated grape-variety/regional sample sets (adjusted *p* < 0.05), but no statistically detectable differences associated with harvest stage or the sample set × harvest stage interaction were observed. No within-origin harvest-stage-related differences were detected.

For α-terpineol, the exploratory two-way ANOVA indicated differences associated with sample set (adjusted *p* < 0.001) and harvest stage (adjusted *p* < 0.01), without a statistically detectable interaction. In the Blaufränkisch/Mittelburgenland sample set, concentrations tended to be higher at EHD than at later harvest stages, with harvest-stage-related differences in LBG (*p* = 0.003) and HOR (*p* = 0.025). In the Pinot Noir/Thermenregion sample set, harvest-stage-related differences were also observed, but the patterns differed between vineyard origins. The lowest concentrations were observed at SHD in GUN (*p* < 0.001), whereas concentrations in OBE were higher at EHD than at LHD (*p* = 0.010).

The compound β-terpineol was detected in Blaufränkisch wines but not in Pinot Noir wines; therefore, inferential statistical evaluation across sample sets was not performed. Within the Blaufränkisch/Mittelburgenland sample set, harvest-stage-related differences were observed in both vineyard origins (*p* = 0.001), with lower concentrations at LHD than at EHD and SHD.

Nerol showed differences associated with sample set (Benjamini–Hochberg-adjusted *p* < 0.05), harvest stage (adjusted *p* < 0.01), and their interaction (adjusted *p* < 0.05). No harvest-stage-related differences were observed in the Blaufränkisch/Mittelburgenland sample set. In contrast, nerol concentrations increased towards later harvest stages in the Pinot Noir/Thermenregion sample set, with harvest-stage-related differences in GUN (*p* = 0.012) and OBE (*p* = 0.014).

Citronellol showed differences associated with sample set (adjusted *p* < 0.001) and harvest stage (adjusted *p* < 0.05), without a statistically detectable interaction. Within-origin harvest-stage-related differences were observed in LBG (*p* = 0.005) and OBE (*p* = 0.037), with the highest concentrations at SHD.

Geraniol showed no statistically detectable differences associated with sample set, harvest stage, or their interaction in the exploratory two-way ANOVA. Within-origin differences were nevertheless detected in LBG (*p* = 0.007) and GUN (*p* = 0.042), although the direction of the response differed between these vineyard origins.

The calculated sum of free monoterpenes differed between the investigated grape-variety/regional sample sets (adjusted *p* < 0.001), whereas no statistically detectable main difference associated with harvest stage was observed. However, a sample set × harvest stage interaction remained statistically detectable after adjustment (adjusted *p* < 0.05). Within-origin analyses showed harvest-stage-related differences only in LBG (*p* = 0.003), where EHD showed higher total free monoterpene concentrations than SHD and LHD.

Overall, free monoterpenes showed highly compound-specific and grape-material-dependent harvest-stage-related patterns. Several free monoterpenes decreased from EHD towards later harvest stages in the Blaufränkisch/Mittelburgenland sample set, whereas individual compounds, such as nerol, increased in the Pinot Noir/Thermenregion sample set. Other compounds showed non-linear patterns, isolated within-origin differences or incomplete quantifiability across the investigated grape materials. These results indicate that the evolution of free monoterpenes cannot be described by a uniform maturity-related trend across all compounds or investigated grape materials.

### 3.5. Free C_13_-Norisoprenoids

The results for free C_13_-norisoprenoids are summarised in Table 10.

Free C_13_-norisoprenoids showed pronounced compound-specific and partly non-linear harvest-stage-related patterns. In the exploratory two-way ANOVA, vitispirane, β-ionone, β-damascenone, and the calculated sum of free C_13_-norisoprenoids showed differences associated with the investigated sample set. Harvest-stage-associated differences were detected for vitispirane, β-ionone, and β-damascenone, whereas no statistically detectable main harvest-stage difference was observed for the calculated sum. Sample set × harvest stage interactions were statistically detectable for vitispirane, β-damascenone, and the calculated sum of free C_13_-norisoprenoids, but not for β-ionone. For TDN, the exploratory two-way ANOVA was not applicable because quantifiable values were not available across all factor levels required for the model.

Vitispirane showed differences associated with sample set and harvest stage (Benjamini–Hochberg-adjusted *p* < 0.001 for both terms), together with a statistically detectable sample set × harvest stage interaction (adjusted *p* < 0.05). Concentrations generally decreased from EHD towards later harvest stages, although the magnitude and exact pattern differed between the investigated grape materials. Within-origin analyses showed harvest-stage-related differences in all vineyard origins, with decreasing patterns in LBG (*p* < 0.001), HOR (*p* = 0.006), GUN (*p* < 0.001), and OBE (*p* < 0.001).

For TDN, within-origin analyses showed harvest-stage-related differences in HOR (*p* = 0.001) and OBE (*p* < 0.001), whereas no such difference was observed in LBG (*p* > 0.05). In GUN, TDN was quantifiable at EHD and SHD, but not detected at LHD; therefore, no within-origin inferential test was performed for this vineyard origin. Overall, TDN concentrations were low across the investigated wines and showed vineyard-origin-specific harvest-stage-related patterns rather than a uniform response across all investigated grape materials.

For β-ionone, the exploratory two-way ANOVA showed differences associated with sample set and harvest stage (adjusted *p* < 0.001 for both terms), whereas the sample set × harvest stage interaction was not statistically detectable after adjustment (adjusted *p* = 0.105). Within-origin analyses indicated harvest-stage-related differences in all vineyard origins. Concentrations generally decreased from EHD towards SHD and LHD, although the magnitude and exact pattern of this decrease differed among vineyard origins.

For β-damascenone, the exploratory two-way ANOVA showed differences associated with sample set (adjusted *p* < 0.001), harvest stage (adjusted *p* < 0.01), and their interaction (adjusted *p* < 0.01). In contrast to vitispirane and β-ionone, β-damascenone did not show a uniform decreasing pattern. In the Blaufränkisch/Mittelburgenland sample set, harvest-stage-related differences were observed only in LBG (*p* = 0.002), where LHD showed lower concentrations than EHD and SHD. In the Pinot Noir/Thermenregion sample set, harvest-stage-related differences were observed in both vineyard origins, with the highest concentrations at SHD in GUN (*p* < 0.001) and at SHD or LHD in OBE (*p* = 0.001).

The calculated sum of free C_13_-norisoprenoids differed between the investigated sample sets (adjusted *p* < 0.001), whereas no statistically detectable main difference associated with harvest stage was observed (adjusted *p* = 0.069). However, the sample set × harvest stage interaction remained statistically detectable after adjustment (adjusted *p* < 0.01), indicating that the overall harvest-stage response differed between the investigated sample sets. In the Blaufränkisch/Mittelburgenland sample set, total concentrations tended to be highest at EHD, with harvest-stage-related differences in LBG (*p* < 0.001) and HOR (*p* = 0.005). In the Pinot Noir/Thermenregion sample set, total concentrations were highest at SHD, with harvest-stage-related differences in GUN (*p* = 0.002) and OBE (*p* = 0.005).

Overall, free C_13_-norisoprenoids showed compound-specific and grape-material-dependent harvest-stage-related patterns. Vitispirane and β-ionone generally decreased with advancing harvest stage, whereas β-damascenone showed a more complex response, particularly in the Pinot Noir/Thermenregion sample set. The TDN concentrations were low and could not be evaluated across all factor levels. These results indicate that the evolution of free C_13_-norisoprenoids in the bottled wines cannot be described by a uniform maturity-related trend across all compounds or investigated grape materials.

### 3.6. Principal Component Analysis

**Figure 1:**
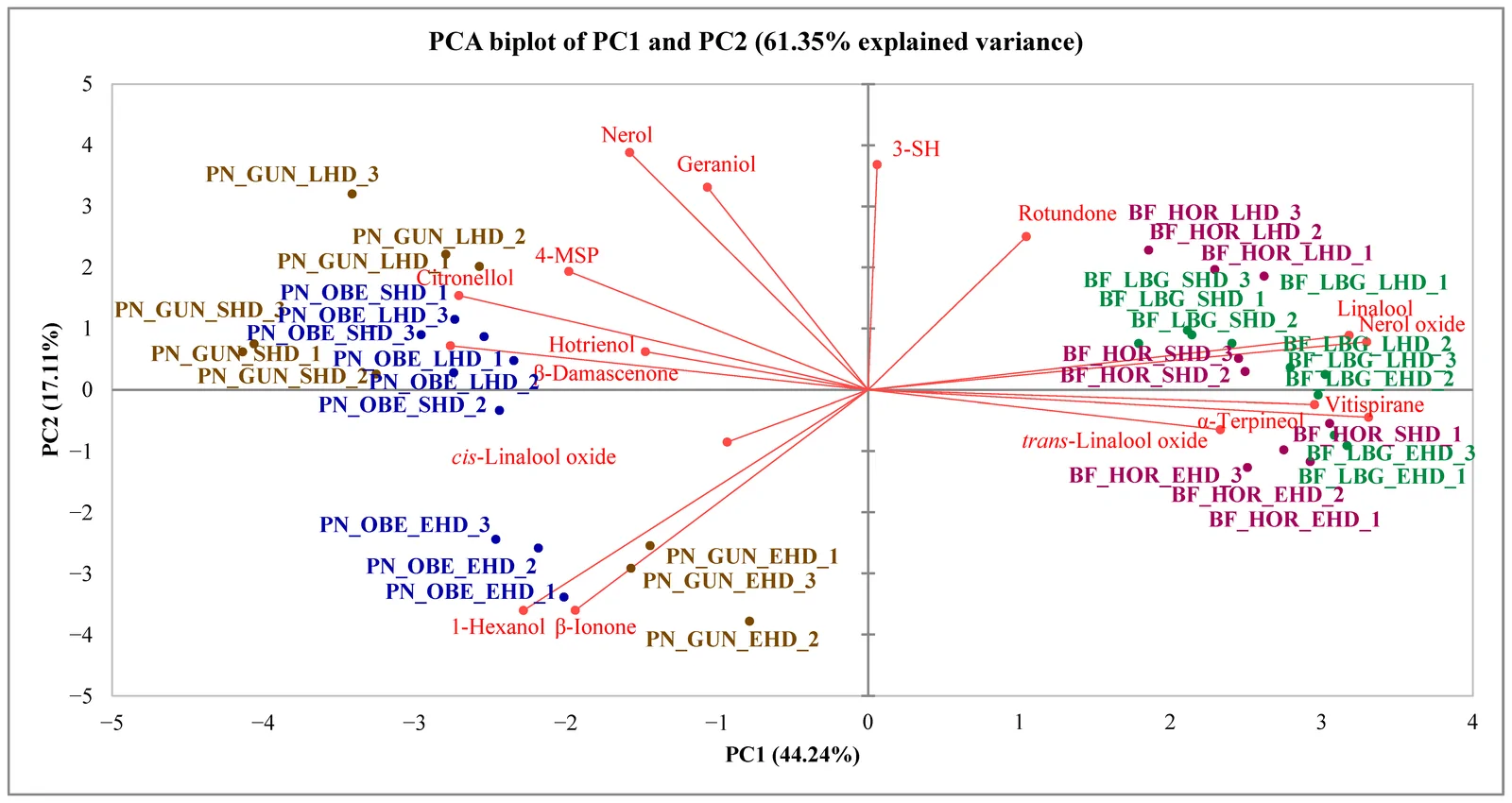
Principal component analysis (PCA) biplot of the aroma-related volatile compounds included in the analysis. The PCA was performed on the correlation matrix of log10-transformed concentration data using only compounds with quantifiable values across all samples. The first two principal components explained 61.35% of the total variance (PC1, 44.24%; PC2, 17.11%). Samples are labelled according to grape-variety/regional sample set, vineyard origin, harvest stage, and replicate number. Abbreviations: BF, Blaufränkisch; PN, Pinot Noir; LBG, Lutzmannsburg; HOR, Horitschon; GUN, Guntramsdorf; OBE, Oberwaltersdorf; EHD, early harvest date; SHD, standard harvest date; LHD, late harvest date. Vectors represent the volatile compounds included in the PCA. Vineyard-origin labels are used to identify the experimental grape materials. The PCA was used as an exploratory visualisation tool and is not intended to support direct statistical comparisons between vineyard sites, wine-growing regions, or grape varieties.

Principal component analysis (PCA) was performed as an exploratory multivariate tool to visualise patterns in the aroma-related volatile composition of the investigated wines. The results are presented in Figure 1.

The first two principal components explained 61.35% of the total variance, with 44.24% attributed to PC1 and 17.11% to PC2. The PCA biplot showed a pronounced separation of the investigated grape-variety/regional sample sets along PC1. Pinot Noir/Thermenregion samples were mainly located in the negative region of PC1, whereas Blaufränkisch/Mittelburgenland samples were predominantly positioned in the positive region of PC1. This suggests that the overall volatile profiles differed between the investigated grape materials under the conditions of this study.

Additional structuring according to harvest stage was observed within the sample sets, although this pattern was less pronounced than the separation according to grape material and was not uniform across all vineyard origins. In the Blaufränkisch/Mittelburgenland sample set, EHD samples tended to be positioned at positive PC1 values and lower PC2 values, whereas several SHD and LHD samples were located at higher PC2 values. In the Pinot Noir/Thermenregion sample set, EHD samples, particularly from OBE and partly from GUN, were located in the lower negative PC2 region, while several SHD and LHD samples were positioned at higher PC2 values. This suggests that harvest stage contributed to the multivariate variation in aroma-related volatile composition, but that the direction and extent of this structuring depended on the investigated grape material.

The distribution of compound vectors reflected the compound-specific patterns observed in the univariate analyses. Linalool, nerol oxide, α-terpineol, vitispirane, and rotundone were oriented towards the positive region of PC1 and were therefore mainly associated with the Blaufränkisch/Mittelburgenland sample set. In contrast, 1-hexanol, β-ionone, β-damascenone, hotrienol, citronellol, *cis*-linalool oxide, and 4-MSP were oriented towards the negative region of PC1 and contributed to the differentiation of the Pinot Noir/Thermenregion sample set. The vectors for 3-SH, nerol, and geraniol were mainly oriented towards the positive region of PC2, suggesting that these compounds contributed to variation along the second principal component.

Overall, the PCA was consistent with the univariate results by showing a clear multivariate separation between the investigated grape-variety/regional sample sets, with additional harvest-stage-related structuring within sample sets. These patterns should be interpreted as exploratory associations among samples and compounds under the present experimental conditions, rather than as direct effects of vineyard site, wine-growing region, or grape variety alone.

## 4. Discussion

The Discussion addresses two main questions: first, whether operationally defined maturity-related harvest stage was associated with compound-specific changes in the selected free aroma-related volatile compounds; and second, whether the direction and magnitude of these responses differed between the investigated grape-variety/regional sample sets. Based on previous ripening studies, C_6_ alcohols were expected to decrease with advancing harvest stage, whereas selected free volatile polyfunctional thiols, free monoterpenes, and free C_13_-norisoprenoids were expected to show increasing or non-linear responses. The findings are interpreted within the specific grape materials and conditions of the 2023 vintage, while the implications of the non-fully crossed experimental design are discussed in detail in Section 4.6.

### 4.1. Free Volatile Polyfunctional Thiols

Free volatile polyfunctional thiols showed harvest-stage-related variation, although responses differed between compounds. The concentrations of 4-MSP were generally higher at SHD and LHD than at EHD. This pattern is compatible with a previous report describing changes in thiol-precursor abundance during grape ripening [11]. Accordingly, later maturity-related harvest stages may be associated with higher concentrations of selected volatile thiols in the resulting wines.

For 3-SH, the exploratory two-way ANOVA indicated harvest-stage-associated differences, whereas neither a main difference between the investigated sample sets nor a sample set × harvest stage interaction was statistically detectable after Benjamini–Hochberg adjustment. The increase in 3-SH concentrations with advancing harvest stage is compatible with a previous study describing changes in S-cysteinylated and S-glutathionylated thiol precursors during grape ripening [11]. The pronounced harvest-stage differentiation observed in the Pinot Noir material is also compatible with reports documenting potent volatile thiols or their precursors in Pinot Noir materials [38,39].

The ester 3-SHA was not detected in any treatment, which is plausible because thiol acetates often occur at very low concentrations in red wines and may remain below analytical detection limits depending on matrix composition, yeast metabolism, and storage conditions [15]. Overall, maturity-related harvest timing was associated with changes in thiol-related volatile composition, particularly through changes in 3-SH, but the response was compound-specific and dependent on the investigated grape material. Because cysteinylated and glutathionylated precursor conjugates were not quantified, these patterns refer exclusively to the final volatile thiol concentrations in the bottled wines and cannot distinguish maturity-related changes in grape precursor abundance from differences in extraction, fermentation-related conversion, or subsequent compound stability [40].

### 4.2. C_6_ Alcohols and Their Relationship with Thiols

The C_6_ alcohol results showed a clear harvest-stage-related pattern for 1-hexanol, whereas *cis*-3-hexen-1-ol was detected only at low concentrations and *trans*-2-hexen-1-ol was not detected. The decrease in 1-hexanol from EHD to SHD and LHD is consistent with previous studies reporting higher concentrations of C_6_ aldehydes and alcohols in less mature berries and declining concentrations during ripening [4,5]. Within the lipoxygenase–hydroperoxide lyase pathway, linoleic and α-linolenic acids are converted into C_6_ aldehydes, which can subsequently be reduced by alcohol dehydrogenases to the corresponding C_6_ alcohols. Developmental changes in substrate availability and in the expression or activity of LOX-, HPL-, and ADH-related steps may therefore contribute to the observed decline in 1-hexanol, although these upstream metabolites and enzymes were not measured in the present study [6,41].

Because C_6_ alcohols are associated with green and herbaceous sensory impressions, lower 1-hexanol concentrations at later harvest stages may indicate a reduced contribution of C_6_-alcohol-related green aroma markers in the resulting wines. However, this interpretation should be made cautiously, as no sensory evaluation was performed and the response differed among vineyard origins. In contrast, *cis*-3-hexen-1-ol occurred close to the lower quantifiable concentration range and did not show a robust harvest-stage-related pattern.

The weak negative pooled correlation between 3-SH and 1-hexanol should be interpreted as co-variation within the present dataset rather than as evidence of a direct metabolic conversion or causal relationship. One documented route to 3-SH precursors involves the reaction of glutathione with (E)-2-hexenal and the formation of glutathionylated intermediates, whereas 1-hexanol is a saturated C_6_ alcohol and is not itself a specific marker of this precursor-forming pathway [42]. Moreover, the concentration of 3-SH in the resulting wine depends not only on grape-derived precursor abundance, but also on grape processing and on yeast-mediated precursor transport, intracellular processing, and β-lyase activity during alcoholic fermentation [43]. Because (E)-2-hexenal, thiol conjugates and yeast-related conversion steps were not quantified, the observed weak correlation may primarily reflect the opposite associations of 1-hexanol and 3-SH with harvest stage or differences among the investigated grape materials. It therefore cannot be used as evidence for a direct biochemical relationship between the two measured compounds.

### 4.3. Rotundone

Rotundone showed a different pattern from most other investigated compounds. In the exploratory two-way ANOVA, no statistically detectable differences associated with grape-variety/regional sample set, harvest stage, or their interaction were observed. Thus, no consistent overall response of rotundone concentrations to harvest timing was detected across the investigated wines.

Within individual vineyard origins, however, harvest-stage-related differences were observed. In some cases, higher concentrations were found at LHD, which is consistent with reports describing rotundone accumulation during later stages of grape ripening [24,27,44]. Rotundone is mainly localised in berry skins and is generally reported to accumulate after véraison, often increasing towards harvest [24,28].

This pattern was not consistent across all wines. Non-linear trends, including maximum concentrations at SHD in one Pinot Noir vineyard origin, indicate that rotundone concentration in wine cannot be explained by harvest stage alone. Previous studies have shown that rotundone formation is affected by berry development, skin metabolism, temperature, and water status [25]. Because these factors could not be separated in the present experimental design, the observed variability should be interpreted as grape-material-specific variation within the investigated dataset.

The absence of a statistically detectable sample-set-associated difference may also reflect the moderate rotundone potential of the investigated grape materials. Although pronounced varietal differences in rotundone have been reported [24,45], the concentrations measured in the investigated wines were low to moderate compared with values typically reported for strongly peppery cultivars such as Syrah or Duras [25]. Overall, the present data do not support a generalisable harvest-stage effect for rotundone across all investigated wines.

### 4.4. Free Monoterpenes

Free monoterpenes showed pronounced compound-specific variation, with several compounds differing between the investigated grape-variety/regional sample sets and/or among harvest stages. However, the response patterns were often non-linear and not uniform across the compound class. Differences associated with the investigated sample sets were observed for several compounds, including linalool, α-terpineol, and nerol oxide, as well as for the calculated sum of free monoterpenes. The compound β-terpineol was detected only in the Blaufränkisch material, while *trans*-limonene oxide was not quantifiable across all required factor levels. These findings are consistent with cultivar-related variation in terpene biosynthesis, which has been linked to differences in terpene synthase repertoires and precursor formation [16,46,47]. In the present study, however, this variation should be interpreted within the investigated grape-variety/regional sample sets.

Several free monoterpenes occurred at higher concentrations in the Blaufränkisch/Mittelburgenland material, whereas some compounds were low or not detected in the Pinot Noir/Thermenregion material. This is consistent with the generally subtle terpene expression reported for many non-aromatic red cultivars, including Pinot Noir [3]. However, the present results also show that free monoterpenes cannot be treated as a uniform chemical group, as individual compounds responded differently to harvest timing.

The compound-specific and partly non-linear patterns are biochemically plausible because the measured free monoterpene pool represents the balance of several simultaneous processes rather than a single uniform ripening response. The biosynthesis and glycosylation of individual monoterpenes are associated with different terpene synthase and glycosyltransferase genes, whose expression and relationship with metabolite accumulation may differ among compounds, cultivars, and ripening stages [18]. Consequently, increased biosynthesis or accumulation of a glycosidically bound precursor pool does not necessarily result in a parallel increase in the corresponding free monoterpene. Following grape processing, the free fraction may be further affected by precursor hydrolysis and by compound-specific rearrangement or degradation during fermentation and bottle storage [48]. These parallel processes of formation, glycosylation, release, and transformation provide a mechanistic explanation for why some free monoterpenes reached their highest concentrations at EHD or SHD, whereas others showed little variation or different harvest-stage responses. Because only free monoterpenes in bottled wines were quantified, the present data cannot determine which of these individual processes caused each observed pattern. Combined analysis of free and glycosidically bound fractions would be required to distinguish changes in biosynthesis and precursor accumulation from release and subsequent transformation.

### 4.5. Free C_13_-Norisoprenoids

Free C_13_-norisoprenoids showed pronounced compound-specific and partly non-linear responses to harvest stage. In the exploratory two-way ANOVA, statistically detectable harvest-stage-associated differences were observed for vitispirane, β-ionone, and β-damascenone, whereas the calculated sum of free C_13_-norisoprenoids did not show an overall harvest-stage main effect. Sample set × harvest stage interactions remained statistically detectable after Benjamini–Hochberg adjustment for vitispirane, β-damascenone, and the calculated sum of free C_13_-norisoprenoids, whereas the interaction for β-ionone was not statistically detectable. For TDN, the exploratory two-way ANOVA was not applicable because quantifiable values were not available across all required factor levels.

Vitispirane and β-ionone generally decreased with advancing harvest stage in many of the investigated wines. The TDN concentrations were low and showed vineyard-origin-specific patterns rather than a uniform response. At first glance, decreasing or non-uniform patterns for some free C_13_-norisoprenoids may appear to contrast with studies reporting increasing norisoprenoid precursors or norisoprenoid-related aroma potential during grape ripening [2,3]. However, the present study quantified free norisoprenoids in bottled wines rather than total or glycosidically bound norisoprenoid precursors in grapes. Free norisoprenoid concentrations in wine are shaped not only by grape precursor availability, but also by release, transformation, and degradation reactions during vinification and bottle storage.

Recent work has also shown that the ripening dynamics of individual norisoprenoids and their precursors are not uniform. Scharf et al. [31], for example, reported increasing total vitispirane and TDN concentrations until LHD, while total β-damascenone reached a maximum earlier and remained relatively stable during the final ripening stages. In contrast, total β-ionone showed a decreasing trend during further ripening. Such findings support the interpretation that norisoprenoid evolution depends strongly on the individual compound and on the balance between precursor formation, release, and degradation.

A particularly complex pattern was observed for β-damascenone. In the Pinot Noir/Thermenregion material, the highest concentrations were observed at SHD or at later harvest stages, whereas the Blaufränkisch/Mittelburgenland material showed no uniform increase with advancing harvest stage. Such non-linear behaviour is consistent with studies indicating that β-damascenone does not necessarily increase continuously until over-ripeness, but may stabilise or decline after reaching a maturity-related maximum [22,23]. Matrix-related factors such as acidity, pH, and alcohol concentration may additionally influence release, stability, and partitioning mechanisms.

The calculated sum of free C_13_-norisoprenoids further underlines the relevance of this compound class for differentiating the investigated wines, but should be interpreted carefully because it combines compounds with different biochemical origins, release mechanisms, and maturity-related trajectories. Overall, the data do not support a simple maturity model in which all free C_13_-norisoprenoids increase with later harvest. Instead, their concentrations in bottled wine appeared to depend on the individual compound, the investigated grape material, and the transformation of bound precursors into free aroma compounds during vinification and storage.

### 4.6. Multivariate Interpretation, Practical Implications and Limitations

The PCA was consistent with the univariate results by showing a clear multivariate separation between the investigated Blaufränkisch/Mittelburgenland and Pinot Noir/Thermenregion sample sets, with additional structuring according to harvest stage. The first two principal components explained 61.35% of the total variance. Separation along PC1 was mainly associated with differences between grape-material backgrounds, whereas PC2 reflected additional variation partly related to harvest stage.

The analysed compound classes contributed differently to sample differentiation. Linalool, nerol oxide, α-terpineol, vitispirane, and rotundone were associated with the positive region of PC1 and mainly contributed to the differentiation of the Blaufränkisch/Mittelburgenland sample set. In contrast, 1-hexanol, β-ionone, β-damascenone, hotrienol, citronellol, *cis*-linalool oxide, and 4-MSP were oriented towards the negative region of PC1 and contributed to the differentiation of the Pinot Noir/Thermenregion sample set. The vectors for 3-SH, nerol, and geraniol were mainly oriented towards the positive region of PC2. These multivariate patterns support the interpretation that the investigated aroma-related volatile composition differed mainly according to grape-material background, with additional harvest-stage-related structuring.

From an applied perspective, the results indicate that harvest timing may contribute to changes in selected aroma-related volatile compounds in red wines. Later harvest stages were generally associated with lower concentrations of the C_6_ alcohol 1-hexanol and higher concentrations of 3-SH, suggesting a shift towards lower C_6_-alcohol-related green aroma markers and higher thiol-related volatile concentrations. However, these implications remain chemical in nature, as no sensory evaluation was conducted. Free monoterpenes, rotundone, and free C_13_-norisoprenoids showed more complex and often non-linear responses, indicating that the harvest stage associated with maximum concentrations may differ among compound classes and individual analytes. Accordingly, the present data do not support a general recommendation to delay harvest solely to increase the concentrations of aroma-related volatile compounds. Potential advantages, such as lower 1-hexanol and higher 3-SH concentrations at later harvest stages, must be weighed against concomitant changes in the basic wine matrix. In several of the investigated grape materials, particularly the Pinot Noir materials, later harvest stages were accompanied by lower titratable acidity and higher pH, while final alcohol concentrations also increased in some vineyard origins despite sucrose addition towards a target of 25.0 °Brix where required. Moreover, several free monoterpenes, free C_13_-norisoprenoids, and rotundone reached their highest concentrations at EHD or SHD rather than consistently at LHD. The practical harvest optimum should therefore be regarded as compound-, grape-material- and production-objective-specific rather than as uniformly associated with delayed harvest.

Published odour thresholds provide only approximate context for the potential sensory relevance of the measured concentrations. The concentrations of 4-MSP and 3-SH in the investigated wines were above perception thresholds reported for these compounds in wine or hydroalcoholic model solutions [49,50], whereas rotundone concentrations spanned values below and above the reported detection threshold of approximately 16 ng/L in red wine [51]. However, these comparisons do not demonstrate that the concentration differences among harvest stages were perceptible. Published thresholds are dependent on the matrix, sensory method, and investigated panel and generally describe detection relative to a reference matrix rather than the minimum perceptible difference between two complex wines. In addition, interactions among volatile compounds and between volatile and non-volatile matrix components may modify their sensory expression. Therefore, the present chemical data suggest potential sensory relevance for selected compounds but cannot demonstrate harvest-stage-related sensory differences without direct sensory evaluation.

Several limitations of the experimental design should be considered when interpreting the results. The study was conducted in a single vintage and was based on grape material from four commercial vineyard origins. Grape variety, wine-growing region, vineyard origin, vine age, clone, rootstock, soil conditions, and local microclimatic factors were not fully crossed or experimentally disentangled. The climatic data were included solely to describe the environmental conditions at the investigated vineyard origins; owing to the experimental design, causal effects of individual climatic parameters on the measured volatile compounds cannot be inferred. Consequently, differences between the investigated Blaufränkisch and Pinot Noir wines should not be interpreted as general varietal effects, or as direct effects of vineyard site or wine-growing region alone, but as differences between the investigated grape-variety/regional sample sets under the specific conditions of the 2023 vintage. In addition, harvest date was used as a practical experimental factor, whereas grape maturity was documented by must parameters such as °Brix, pH, and titratable acidity. Although sucrose was added where required towards a target of 25.0 °Brix to reduce differences in fermentable sugar concentration among harvest stages, the musts were not adjusted to an entirely identical starting value and final alcohol concentrations were not fully homogenised. Residual differences in alcohol content and wine matrix composition may have influenced extraction, yeast metabolism, volatile partitioning, and, potentially, the measured concentrations of some aroma-related compounds. Therefore, the observed patterns should be interpreted as the combined outcome of the operationally defined harvest stages, standardised processing conditions, and resulting wine matrices, rather than as effects of grape maturity alone. Bound aroma precursors were not quantified, and no sensory evaluation was performed. Therefore, the results describe changes in selected free aroma-related volatile compounds in bottled wines and do not allow direct conclusions regarding sensory quality or aroma perception.

Despite these limitations, the study provides insight into harvest-stage-related changes in selected free aroma-related volatile compounds within the investigated Blaufränkisch and Pinot Noir grape materials under standardised microvinification conditions. Future studies should include fully crossed multi-vintage experimental designs, larger numbers of vineyard replicates, additional vineyard and maturity descriptors, precursor analyses, and sensory or odour-activity-based evaluation to clarify how grape maturity, genotype, and vineyard background jointly influence red wine volatile composition and its sensory relevance.

## 5. Conclusions

Within the limitations of a one-vintage, non-fully crossed grape-material design, the present study suggests that operationally defined maturity-related harvest timing was associated with changes in selected free aroma-related volatile compounds in wines produced from the investigated Blaufränkisch and Pinot Noir grape materials under standardised microvinification conditions. The observed responses were compound-specific and depended on the investigated grape-variety/regional sample sets.

The C_6_ alcohol 1-hexanol generally decreased with advancing harvest stage, whereas the free volatile polyfunctional thiol 3-SH tended to increase across several vineyard origins. In contrast, free monoterpenes, free C_13_-norisoprenoids, and rotundone showed more complex, partly non-linear and grape-material-dependent patterns, indicating that their concentrations in bottled wines cannot be described by a uniform maturity-related trend. Sample set × harvest stage interactions observed for several compounds further indicated that harvest-stage responses differed between the investigated grape materials.

From a practical perspective, these findings do not support a general recommendation to delay harvest in order to maximise aroma-related volatile compounds. Harvest decisions should instead balance compound-specific volatile responses against concurrent changes in acidity, pH, and alcohol concentration and should be adapted to the specific grape material and production objective.

The PCA was consistent with the univariate results by showing differentiation mainly according to grape-material background, with additional structuring related to harvest stage. However, because grape variety, vineyard origin, vine age, clone, rootstock, and regional background were not fully disentangled in the experimental design, these patterns should not be interpreted as general effects of grape variety, vineyard site, or wine-growing region alone.

Overall, within the specific grape materials and experimental conditions investigated, the findings support harvest timing as a practical factor associated with changes in selected aroma-related volatile compounds in the resulting wines. However, statistically detectable concentration differences do not necessarily correspond to perceptible sensory differences, and the absence of direct sensory evaluation precludes conclusions regarding aroma perception or sensory quality. Future studies using fully crossed multi-vintage designs, additional vineyard and maturity descriptors, glycosidically bound precursors, and sensory or odour-activity-based evaluation are required to clarify the combined effects of grape maturity, genotype and vineyard background and to assess the sensory relevance of the observed chemical differences.

## Figures and Tables

**Table 1:** Characterisation of the experimental vineyards.

Vineyard Origin	Lutzmannsburg, Riede Alt Satz	Horitschon, Riede Lauspil	Guntramsdorf, Riede Eichkogel	Oberwaltersdorf, Riede Kräutergarten
**Grape variety**	Blaufränkisch		Pinot Noir	
**Coordinates**	47°27′02″ N 16°38′45″ E	47°34′52″ N 16°33′11″ E	48°03′36″ N 16°17′25″ E	47°58′02″ N 16°19′51″ E
**Year planted**	2003	1990	2000	2000
**Rootstock**	Kober 5BB		Fercal	
**Clone**	Selection IBY		Dijon 115	FR 52/86
**Elevation**	261 m	243 m	295 m	211 m
**Plant protection**	Integrated			
**Soil management and general vineyard management**	Pruning, training system, soil cultivation, and under-vine management: **standard commercial practice**			

**Table 2:** Climatic conditions at the investigated vineyard origins during the 2023 growing and experimental harvest periods.

Grape Variety	Vineyard Origin	GDD_10_, 1 Apr–LHD [°C d]	Huglin Index, 1 Apr–30 Sep [°C d]	Mean TM24, 1 Apr–LHD [°C]	RR, 1 Apr–LHD [mm]	Mean TM24, EHD–LHD [°C]	Mean TN, EHD–LHD [°C]	Mean TX, EHD–LHD [°C]	RR, EHD–LHD [mm]
**Blaufränkisch**	**Lutzmannsburg**	1560	2069	17.7	537.0	17.6	12.2	24.3	24.3
**Blaufränkisch**	**Horitschon**	1632	2132	18.0	490.8	18.1	12.8	24.5	24.2
**Pinot Noir**	**Guntramsdorf**	1437	1973	17.2	401.3	18.4	11.8	25.5	16.3
**Pinot Noir**	**Oberwaltersdorf**	1567	2108	18.0	402.1	19.1	12.4	26.4	15.2

Daily meteorological data were obtained from the SPARTACUS-v3 dataset of GeoSphere Austria at 1-km spatial resolution using the coordinates of the respective vineyard origins [29]. Abbreviations: TM24, 24-h mean air temperature; TN, daily minimum air temperature; TX, daily maximum air temperature; RR, precipitation sum; EHD, early harvest date; LHD, late harvest date. Calculation periods were inclusive. Growing degree days above a base temperature of 10 °C (GDD_10_) were calculated as the sum of max(TM24 − 10 °C, 0) from 1 April to the respective LHD. The Huglin Index was calculated from 1 April to 30 September using TM24, TX and a latitude coefficient of 1.05; negative daily contributions were set to zero [30].

**Table 3:** Harvest dates of the grapes (EHD = early harvest date; SHD = standard harvest date; LHD = late harvest date).

Grape Variety	Vineyard Origin	Harvest Dates		
		EHD	SHD	LHD
**Blaufränkisch**	**Lutzmannsburg**	15 September 2023	29 September 2023	13 October 2023
	**Horitschon**	15 September 2023	29 September 2023	13 October 2023
**Pinot Noir**	**Guntramsdorf**	8 September 2023	22 September 2023	6 October 2023
	**Oberwaltersdorf**	8 September 2023	22 September 2023	6 October 2023

**Table 4:** Quantified must parameters.

Must Parameter	Variety	Vineyard Origin	Experimental Variants		
			EHD (*n* = 1)	SHD (Mean ± SD, *n* = 3)	LHD (Mean ± SD, *n* = 3)
Density [g/cm^3^]	BF	LBG	1.0743	1.0771 ± 0.0047	1.0878 ± 0.0001
		HOR	1.0806	1.0861 ± 0.0001	1.0920 ± 0.0001
	PN	GUN	1.0840	1.0935 ± 0.0002	1.1091 ± 0.0001
		OBE	1.0792	1.0918 ± 0.0003	1.1011 ± 0.0002
Glucose [g/L]	BF	LBG	90.2	89.5 ± 6.6	103.8 ± 0.3
		HOR	100.4	102.6 ± 0.7	109.9 ± 0.4
	PN	GUN	100.3	119.0 ± 0.4	130.3 ± 0.6
		OBE	92.4	115.6 ± 0.8	117.8 ± 0.6
Fructose [g/L]	BF	LBG	92.2	93.4 ± 6.5	110.2 ± 0.5
		HOR	101.0	106.4 ± 0.2	115.5 ± 0.5
	PN	GUN	102.7	124.6 ± 0.1	139.3 ± 0.7
		OBE	96.9	121.7 ± 1.1	130.3 ± 0.9
°Brix	BF	LBG	17.6	18.7 ± 0.1	20.6 ± 0.1
		HOR	19.1	20.1 ± 0.1	21.6 ± 0.1
	PN	GUN	19.4	22.5 ± 0.0	25.3 ± 0.1
		OBE	18.4	22.1 ± 0.1	23.5 ± 0.1
Titratable acidity, expressed as tartaric acid [g/L]	BF	LBG	8.3	8.0 ± 0.2	7.4 ± 0.0
		HOR	7.4	8.1 ± 0.0	7.5 ± 0.0
	PN	GUN	8.4	3.7 ± 0.1	5.9 ± 0.1
		OBE	8.6	5.1 ± 0.1	6.2 ± 0.0
pH	BF	LBG	2.96	3.24 ± 0.03	3.15 ± 0.01
		HOR	3.02	3.22 ± 0.01	3.20 ± 0.01
	PN	GUN	3.08	3.08 ± 0.01	3.37 ± 0.05
		OBE	3.15	3.04 ± 0.00	3.47 ± 0.02
Tartaric acid [g/L]	BF	LBG	6.9	7.5 ± 0.1	7.3 ± 0.1
		HOR	6.0	7.7 ± 0.1	7.4 ± 0.1
	PN	GUN	8.6	3.4 ± 0.2	7.5 ± 0.1
		OBE	7.9	4.2 ± 0.2	7.5 ± 0.1
Malic acid [g/L]	BF	LBG	3.3	3.4 ± 0.1	2.5 ± 0.1
		HOR	3.1	3.3 ± 0.0	2.7 ± 0.1
	PN	GUN	2.9	0.9 ± 0.0	1.8 ± 0.1
		OBE	3.6	1.8 ± 0.0	2.3 ± 0.0
Volatile acidity [g/L]	BF	LBG	0.1	0.2 ± 0.0	0.3 ± 0.0
		HOR	0.2	0.1 ± 0.0	0.3 ± 0.0
	PN	GUN	0.1	0.3 ± 0.0	0.3 ± 0.0
		OBE	0.1	0.3 ± 0.0	0.2 ± 0.0
NOPA [mg/L]	BF	LBG	177.5	219.3 ± 6.4	165.3 ± 4.8
		HOR	172.5	195.7 ± 6.8	163.0 ± 6.6
	PN	GUN	154.0	163.8 ± 4.6	160.3 ± 2.8
		OBE	188.5	188.2 ± 1.0	202.8 ± 4.6
Ammonium [mg/L]	BF	LBG	134.0	139.3 ± 2.9	92.2 ± 7.2
		HOR	127.5	126.5 ± 6.9	89.5 ± 5.8
	PN	GUN	123.0	85.8 ± 3.3	65.8 ± 7.9
		OBE	130.5	105.3 ± 1.6	91.3 ± 7.3
YAN (calculated) [mg/L]	BF	LBG	287.7	333.9 ± 5.3	241.1 ± 9.8
		HOR	277.4	299.7 ± 11.6	236.6 ± 9.7
	PN	GUN	255.2	234.4 ± 5.5	214.5 ± 4.6
		OBE	295.8	274.8 ± 2.4	278.0 ± 9.0

Values refer to musts before chaptalisation. Where applicable, for concentration parameters expressed in g/L, the limit of detection was 0.1 g/L and the limit of quantification was 0.2 g/L. Abbreviations: NOPA, α-amino nitrogen; YAN, yeast-assimilable nitrogen; BF, Blaufränkisch; PN, Pinot Noir; LBG, Lutzmannsburg; HOR, Horitschon; GUN, Guntramsdorf; OBE, Oberwaltersdorf; EHD, early harvest date; SHD, standard harvest date; LHD, late harvest date. Exact harvest dates are given in Table 3. For EHD, must parameters were determined from one pooled must sample prepared from the three replicate grape lots and are therefore reported without a standard deviation. For SHD and LHD, values are presented as mean ± SD of the three replicate grape lots. The EHD must parameters were used descriptively to document the maturity status of the grape material at processing; aroma analyses and wine parameters were based on independently vinified biological replicates.

**Table 5:** Quantified wine parameters.

Wine Parameter	Variety	Vineyard Origin	Experimental Variants (Mean ± SD, *n* = 3)		
			EHD	SHD	LHD
Density [g/cm^3^]	BF	LBG	0.9921 ± 0.0004	0.9907 ± 0.0010	0.9926 ± 0.0003
		HOR	0.9912 ± 0.0011	0.9905 ± 0.0014	0.9934 ± 0.0003
	PN	GUN	0.9912 ± 0.0001	0.9894 ± 0.0004	0.9911 ± 0.0003
		OBE	0.9911 ± 0.0002	0.9932 ± 0.0004	0.9918 ± 0.0001
Alcohol [% vol.]	BF	LBG	14.3 ± 0.0	14.7 ± 0.2	14.8 ± 0.0
		HOR	15.2 ± 0.2	14.9 ± 0.3	14.4 ± 0.2
	PN	GUN	14.4 ± 0.0	15.5 ± 0.2	16.1 ± 0.1
		OBE	14.2 ± 0.0	14.5 ± 0.1	15.3 ± 0.2
Glucose [g/L]	BF	LBG	n.q.	n.q.	n.q.
		HOR	n.q.	n.q.	n.q.
	PN	GUN	n.q.	n.q.	n.q.
		OBE	n.q.	n.q.	n.q.
Fructose [g/L]	BF	LBG	n.q.	n.q.	n.q.
		HOR	n.q.	n.q.	n.q.
	PN	GUN	n.q.	n.q.	n.q.
		OBE	n.q.	n.q.	n.q.
Titratable acidity, expressed as tartaric acid [g/L]	BF	LBG	6.5 ± 0.2	5.3 ± 0.1	6.3 ± 0.0
		HOR	6.3 ± 0.1	5.2 ± 0.1	6.0 ± 0.2
	PN	GUN	6.2 ± 0.1	4.3 ± 0.1	4.4 ± 0.1
		OBE	6.1 ± 0.2	4.9 ± 0.1	4.8 ± 0.0
pH	BF	LBG	3.35 ± 0.04	3.43 ± 0.02	3.39 ± 0.02
		HOR	3.41 ± 0.02	3.46 ± 0.03	3.55 ± 0.02
	PN	GUN	3.43 ± 0.01	3.58 ± 0.04	3.65 ± 0.04
		OBE	3.47 ± 0.04	3.56 ± 0.01	3.65 ± 0.01
Tartaric acid [g/L]	BF	LBG	2.6 ± 0.1	0.9 ± 0.1	2.5 ± 0.0
		HOR	2.5 ± 0.1	1.0 ± 0.2	2.3 ± 0.2
	PN	GUN	2.2 ± 0.1	0.8 ± 0.2	0.8 ± 0.0
		OBE	1.8 ± 0.1	0.6 ± 0.1	0.6 ± 0.0
Malic acid [g/L]	BF	LBG	1.3 ± 0.1	1.4 ± 0.0	1.0 ± 0.0
		HOR	1.6 ± 0.1	1.5 ± 0.1	1.2 ± 0.1
	PN	GUN	1.3 ± 0.0	1.2 ± 0.1	1.1 ± 0.0
		OBE	1.4 ± 0.1	1.4 ± 0.1	1.2 ± 0.0
Lactic acid [g/L]	BF	LBG	n.d.	n.d.	n.d.
		HOR	n.d.	n.d.	n.d.
	PN	GUN	n.q.	n.d.	n.d.
		OBE	n.q.	n.d.	n.d.
Volatile acidity [g/L]	BF	LBG	0.2 ± 0.1	0.4 ± 0.0	0.3 ± 0.0
		HOR	0.1 ± 0.0	0.3 ± 0.1	0.2 ± 0.0
	PN	GUN	0.2 ± 0.0	0.5 ± 0.0	0.7 ± 0.0
		OBE	0.2 ± 0.1	0.3 ± 0.0	0.5 ± 0.0
Citric acid [g/L]	BF	LBG	n.d.	0.1 ± 0.0	n.d.
		HOR	n.d.	0.1 ± 0.0	n.d.
	PN	GUN	0.1 ± 0.0	n.d.	n.d.
		OBE	0.1 ± 0.0	0.2 ± 0.0	n.d.

Where applicable, for concentration parameters expressed in g/L, the limit of detection was 0.1 g/L and the limit of quantification was 0.2 g/L. Abbreviations: n.d., not detected; n.q., detected but below the limit of quantification; BF, Blaufränkisch; PN, Pinot Noir; LBG, Lutzmannsburg; HOR, Horitschon; GUN, Guntramsdorf; OBE, Oberwaltersdorf; EHD, early harvest date; SHD, standard harvest date; LHD, late harvest date. Exact harvest dates are given in Table 3. Values are presented as mean ± SD of three independently harvested and vinified biological replicates. All wines completed alcoholic fermentation, as indicated by non-quantifiable glucose and fructose concentrations. No evidence of malolactic fermentation was observed under the analytical conditions used, as lactic acid was non-detectable or below the limit of quantification.

**Table 6:** Concentrations of selected free volatile polyfunctional thiols in the investigated wines according to sample set, vineyard origin, and harvest stage.

Free Volatile Polyfunctional Thiols [ng/L]	Significance of Effects		Interaction	Sample Set	Vineyard Origin	Experimental Variants (Mean ± SD, *n* = 3)		
	Sample Set (S)	Harvest Stage (H)	S × H			EHD	SHD	LHD
4-MSP	***	**	n.s.	BF	LBG [*p* = **0.001**]	5.4 ^a^ ± 1.7	13.4 ^b^ ± 0.8	13.1 ^b^ ± 1.3
					HOR [*p* > 0.05]	3.7 ± 0.6	3.0 ± 0.7	4.1 ± 0.0
				PN	GUN [*p* < **0.001**]	5.4 ^a^ ± 1.3	12.5 ^b^ ± 1.4	15.1 ^b^ ± 0.7
					OBE [*p* = **0.001**]	7.7 ^a^ ± 0.7	13.4 ^b^ ± 2.3	15.2 ^b^ ± 0.8
3-SH	n.s.	***	n.s.	BF	LBG [*p* = **0.003**]	212.1 ^a^ ± 6.3	210.2 ^a^ ± 6.0	237.4 ^b^ ± 6.1
					HOR [*p* < **0.001**]	143.1 ^a^ ± 7.5	162.8 ^b^ ± 7.5	195.8 ^c^ ± 2.9
				PN	GUN [*p* < **0.001**]	141.8 ^a^ ± 4.9	166.6 ^b^ ± 8.4	239.6 ^c^ ± 7.0
					OBE [*p* < **0.001**]	159.4 ^a^ ± 7.3	185.0 ^b^ ± 4.1	239.0 ^c^ ± 4.6
3-SHA	n.a.	n.a.	n.a.	BF	LBG	n.d.	n.d.	n.d.
					HOR	n.d.	n.d.	n.d.
				PN	GUN	n.d.	n.d.	n.d.
					OBE	n.d.	n.d.	n.d.

Concentrations of the investigated analytes in the analysed wines according to sample set, vineyard origin, and harvest stage. Units are given in the respective table headings. The term “sample set” refers to the grouped Blaufränkisch/Mittelburgenland and Pinot Noir/Thermenregion materials used in the exploratory two-way ANOVA. Abbreviations: BF, Blaufränkisch; PN, Pinot Noir; LBG, Lutzmannsburg; HOR, Horitschon; GUN, Guntramsdorf; OBE, Oberwaltersdorf; EHD, early harvest date; SHD, standard harvest date; LHD, late harvest date. Exact harvest dates are given in Table 3. The corresponding basic must parameters, including °Brix, are presented in Table 4. Values are presented as mean ± SD of three independently harvested and vinified biological replicates. Mean values and standard deviations are rounded for presentation; statistical analyses were performed using unrounded numerical concentration data. Symbols shown for sample set (S), harvest stage (H), and the S × H interaction are based on Benjamini–Hochberg-adjusted *p*-values from the exploratory two-way ANOVA of log10-transformed concentration data. Adjustment was performed separately across the eligible analytical endpoints for each model term. Bold *p*-values in square brackets indicate statistically detectable overall harvest-stage effects within the respective vineyard origin according to one-way ANOVA (*p* < 0.05). Different lowercase letters indicate statistically detectable differences between harvest stages within the same vineyard origin according to one-way ANOVA followed by Tukey’s HSD test (*p* < 0.05). These within-origin comparisons describe harvest-stage-related differences within each grape material and are not intended as direct comparisons between vineyard sites, wine-growing regions, or grape varieties. Symbol levels: ** *p* < 0.01; *** *p* < 0.001; n.s., not significant; n.a., not applicable; n.d., not detected.

**Table 7:** Concentrations of selected C_6_ alcohols in the investigated wines according to sample set, vineyard origin and harvest stage.

C_6_ Alcohols [mg/L]	Significance of Effects		Interaction	Sample Set	Vineyard Origin	Experimental Variants (Mean ± SD, *n* = 3)		
	Sample Set (S)	Harvest Stage (H)	S × H			EHD	SHD	LHD
1-Hexanol	***	***	**	BF	LBG [*p* > 0.05]	1.1 ± 0.1	1.0 ± 0.1	1.0 ± 0.0
					HOR [*p* = **0.012**]	1.1 ^b^ ± 0.0	0.9 ^a^ ± 0.1	0.8 ^a^ ± 0.1
				PN	GUN [*p* < **0.001**]	2.2 ^b^ ± 0.1	1.3 ^a^ ± 0.1	1.2 ^a^ ± 0.1
					OBE [*p* < **0.001**]	2.7 ^b^ ± 0.1	1.7 ^a^ ± 0.1	1.6 ^a^ ± 0.1
*cis*-3-Hexen-1-ol	n.a.	n.a.	n.a.	BF	LBG	n.d.	n.d.	0.1 ± 0.0
					HOR [*p* > 0.05]	0.1 ± 0.1	0.1 ± 0.0	0.1 ± 0.0
				PN	GUN [*p* > 0.05]	0.1 ± 0.0	0.1 ± 0.1	0.1 ± 0.1
					OBE [*p* > 0.05]	0.1 ± 0.0	0.1 ± 0.0	0.1 ± 0.0
*trans*-2-Hexen-1-ol	n.a.	n.a.	n.a.	BF	LBG	n.d.	n.d.	n.d.
					HOR	n.d.	n.d.	n.d.
				PN	GUN	n.d.	n.d.	n.d.
					OBE	n.d.	n.d.	n.d.

Concentrations of the investigated analytes in the analysed wines according to sample set, vineyard origin, and harvest stage. Units are given in the respective table headings. The term “sample set” refers to the grouped Blaufränkisch/Mittelburgenland and Pinot Noir/Thermenregion materials used in the exploratory two-way ANOVA. Abbreviations: BF, Blaufränkisch; PN, Pinot Noir; LBG, Lutzmannsburg; HOR, Horitschon; GUN, Guntramsdorf; OBE, Oberwaltersdorf; EHD, early harvest date; SHD, standard harvest date; LHD, late harvest date. Exact harvest dates are given in Table 3. The corresponding basic must parameters, including °Brix, are presented in Table 4. Values are presented as mean ± SD of three independently harvested and vinified biological replicates. Mean values and standard deviations are rounded for presentation; statistical analyses were performed using unrounded numerical concentration data. Symbols shown for sample set (S), harvest stage (H), and the S × H interaction are based on Benjamini–Hochberg-adjusted *p*-values from the exploratory two-way ANOVA of log10-transformed concentration data. Adjustment was performed separately across the eligible analytical endpoints for each model term. Bold *p*-values in square brackets indicate statistically detectable overall harvest-stage effects within the respective vineyard origin according to one-way ANOVA (*p* < 0.05). Different lowercase letters indicate statistically detectable differences between harvest stages within the same vineyard origin according to one-way ANOVA followed by Tukey’s HSD test (*p* < 0.05). These within-origin comparisons describe harvest-stage-related differences within each grape material and are not intended as direct comparisons between vineyard sites, wine-growing regions, or grape varieties. Symbol levels: ** *p* < 0.01; *** *p* < 0.001; n.a., not applicable; n.d., not detected.

**Table 8:** Concentrations of rotundone in the investigated wines according to sample set, vineyard origin and harvest stage.

Rotundone [ng/L]	Significance of Effects		Interaction	Sample Set	Vineyard Origin	Experimental Variants (Mean ± SD, *n* = 3)		
	Sample Set (S)	Harvest Stage (H)	S × H			EHD	SHD	LHD
Rotundone	n.s.	n.s.	n.s.	BF	LBG [*p* > 0.05]	25.9 ± 5.6	20.8 ± 3.4	25.9 ± 1.3
					HOR [*p* = **0.005**]	9.1 ^a^ ± 0.9	15.3 ^ab^ ± 5.7	25.1 ^b^ ± 2.9
				PN	GUN [*p* < **0.001**]	18.7 ^b^ ± 1.5	11.5 ^a^ ± 0.6	29.6 ^c^ ± 5.7
					OBE [*p* < **0.001**]	6.8 ^a^ ± 0.7	20.8 ^b^ ± 3.1	7.3 ^a^ ± 0.8

Concentrations of the investigated analytes in the analysed wines according to sample set, vineyard origin, and harvest stage. Units are given in the respective table headings. The term “sample set” refers to the grouped Blaufränkisch/Mittelburgenland and Pinot Noir/Thermenregion materials used in the exploratory two-way ANOVA. Abbreviations: BF, Blaufränkisch; PN, Pinot Noir; LBG, Lutzmannsburg; HOR, Horitschon; GUN, Guntramsdorf; OBE, Oberwaltersdorf; EHD, early harvest date; SHD, standard harvest date; LHD, late harvest date. Exact harvest dates are given in Table 3. The corresponding basic must parameters, including °Brix, are presented in Table 4. Values are presented as mean ± SD of three independently harvested and vinified biological replicates. Mean values and standard deviations are rounded for presentation; statistical analyses were performed using unrounded numerical concentration data. Symbols shown for sample set (S), harvest stage (H), and the S × H interaction are based on Benjamini–Hochberg-adjusted *p*-values from the exploratory two-way ANOVA of log10-transformed concentration data. Adjustment was performed separately across the eligible analytical endpoints for each model term. Bold *p*-values in square brackets indicate statistically detectable overall harvest-stage effects within the respective vineyard origin according to one-way ANOVA (*p* < 0.05). Different lowercase letters indicate statistically detectable differences between harvest stages within the same vineyard origin according to one-way ANOVA followed by Tukey’s HSD test (*p* < 0.05). These within-origin comparisons describe harvest-stage-related differences within each grape material and are not intended as direct comparisons between vineyard sites, wine-growing regions, or grape varieties. Symbol level: n.s., not significant.

**Table 9:** Concentrations of selected free monoterpenes in the investigated wines according to sample set, vineyard origin, and harvest stage.

Free Monoterpenes [µg/L]	Significance of Effects		Interaction	Sample Set	Vineyard Origin	Experimental Variants (Mean ± SD, *n* = 3)		
	Sample Set (S)	Harvest Stage (H)	S × H			EHD	SHD	LHD
*trans*-linalool oxide	***	*	n.s.	BF	LBG [*p* > 0.05]	2.1 ± 0.3	1.9 ± 0.1	1.9 ± 0.1
					HOR [*p* > 0.05]	2.1 ± 0.2	1.9 ± 0.2	1.8 ± 0.1
				PN	GUN [*p* > 0.05]	1.8 ± 0.3	1.6 ± 0.0	1.6 ± 0.1
					OBE [*p* > 0.05]	1.7 ± 0.0	1.6 ± 0.2	1.6 ± 0.1
*cis*-linalool oxide	n.s.	n.s.	n.s.	BF	LBG [*p* > 0.05]	0.7 ± 0.1	0.6 ± 0.1	0.7 ± 0.1
					HOR [*p* = **0.009**]	0.7 ^b^ ± 0.0	0.5 ^a^ ± 0.0	0.7 ^b^ ± 0.1
				PN	GUN [*p* > 0.05]	1.0 ± 0.3	0.8 ± 0.1	0.8 ± 0.1
					OBE [*p* > 0.05]	0.6 ± 0.0	0.6 ± 0.0	0.6 ± 0.1
*trans*-limonene oxide	n.a.	n.a.	n.a.	BF	LBG [*p* < **0.001**]	2.3 ^b^ ± 0.1	3.3 ^c^ ± 0.2	1.7 ^a^ ± 0.1
					HOR [*p* < **0.001**]	2.0 ^b^ ± 0.1	2.6 ^c^ ± 0.1	1.5 ^a^ ± 0.0
				PN	GUN	n.d.	n.d.	n.d.
					OBE	0.9 ± 0.1 (*n* = 2)	1.2 ± 0.1	n.d.
Linalool	***	n.s.	*	BF	LBG [*p* = **0.001**]	22.4 ^b^ ± 2.5	13.9 ^a^ ± 0.2	14.3 ^a^ ± 1.3
					HOR [*p* = **0.020**]	32.1 ^b^ ± 4.5	22.8 ^ab^ ± 4.4	19.6 ^a^ ± 2.2
				PN	GUN [*p* > 0.05]	2.8 ± 0.3	3.5 ± 0.7	3.8 ± 0.1
					OBE [*p* > 0.05]	3.0 ± 0.1	2.4 ± 0.2	3.3 ± 0.8
Hotrienol	*	n.s.	n.s.	BF	LBG [*p* > 0.05]	0.6 ± 0.2	0.6 ± 0.0	0.7 ± 0.0
					HOR [*p* > 0.05]	0.6 ± 0.1	0.5 ± 0.1	0.6 ± 0.2
				PN	GUN [*p* > 0.05]	0.7 ± 0.1	0.7 ± 0.1	0.7 ± 0.1
					OBE [*p* > 0.05]	0.6 ± 0.0	0.7 ± 0.2	0.5 ± 0.0
α-terpineol	***	**	n.s.	BF	LBG [*p* = **0.003**]	9.3 ^b^ ± 1.0	6.8 ^a^ ± 0.0	7.2 ^a^ ± 0.2
					HOR [*p* = **0.025**]	11.7 ^b^ ± 0.5	8.4 ^a^ ± 1.5	9.7 ^ab^ ± 0.3
				PN	GUN [*p* < **0.001**]	3.4 ^b^ ± 0.7	0.3 ^a^ ± 0.1	2.3 ^b^ ± 0.1
					OBE [*p* = **0.010**]	2.9 ^b^ ± 0.2	2.5 ^ab^ ± 0.2	2.1 ^a^ ± 0.2
β-terpineol	n.a.	n.a.	n.a.	BF	LBG [*p* = **0.001**]	3.0 ^b^ ± 1.1	2.0 ^b^ ± 0.1	1.0 ^a^ ± 0.1
					HOR [*p* = **0.001**]	1.8 ^b^ ± 0.2	1.3 ^b^ ± 0.1	0.6 ^a^ ± 0.2
				PN	GUN	n.d.	n.d.	n.d.
					OBE	n.d.	n.d.	n.d.
Nerol oxide	***	*	n.s.	BF	LBG [*p* = **0.002**]	3.2 ^c^ ± 0.3	2.3 ^a^ ± 0.1	2.7 ^b^ ± 0.1
					HOR [*p* = **0.001**]	4.0 ^b^ ± 0.1	3.1 ^a^ ± 0.2	3.0 ^a^ ± 0.2
				PN	GUN [*p* > 0.05]	0.3 ± 0.0	0.2 ± 0.0	0.3 ± 0.0
					OBE [*p* > 0.05]	0.2 ± 0.0	0.2 ± 0.0	0.2 ± 0.0
Nerol	*	**	*	BF	LBG [*p* > 0.05]	0.6 ± 0.0	0.7 ± 0.1	0.5 ± 0.1
					HOR [*p* > 0.05]	0.8 ± 0.1	0.8 ± 0.2	1.1 ± 0.1
				PN	GUN [*p* = **0.012**]	0.6 ^a^ ± 0.2	1.2 ^ab^ ± 0.1	1.6 ^b^ ± 0.4
					OBE [*p* = **0.014**]	0.5 ^a^ ± 0.1	0.9 ^b^ ± 0.2	1.0 ^b^ ± 0.1
Citronellol	***	*	n.s.	BF	LBG [*p* = **0.005**]	2.0 ^a^ ± 0.1	2.9 ^b^ ± 0.3	2.3 ^a^ ± 0.1
					HOR [*p* > 0.05]	3.0 ± 0.2	3.0 ± 0.6	3.5 ± 0.3
				PN	GUN [*p* > 0.05]	3.9 ± 1.0	6.0 ± 0.7	5.3 ± 0.9
					OBE [*p* = **0.037**]	3.3 ^a^ ± 0.3	4.7 ^b^ ± 0.9	3.4 ^ab^ ± 0.2
Geraniol	n.s.	n.s.	n.s.	BF	LBG [*p* = **0.007**]	5.9 ^ab^ ± 0.4	6.4 ^b^ ± 0.2	5.2 ^a^ ± 0.2
					HOR [*p* > 0.05]	7.3 ± 0.4	7.8 ± 1.6	9.4 ± 0.6
				PN	GUN [*p* = **0.042**]	5.5 ^a^ ± 1.4	7.8 ^ab^ ± 1.0	9.3 ^b^ ± 1.6
					OBE [*p* > 0.05]	7.0 ± 0.7	7.6 ± 1.3	8.1 ± 0.2
∑ free monoterpenes	***	n.s.	*	BF	LBG [*p* = **0.003**]	52.1 ^b^ ± 5.4	41.1 ^a^ ± 0.1	38.2 ^a^ ± 1.5
					HOR [*p* > 0.05]	66.0 ± 5.5	52.6 ± 8.8	51.4 ± 3.4
				PN	GUN [*p* > 0.05]	20.1 ± 4.4	22.1 ± 1.7	25.7 ± 3.0
					OBE [*p* > 0.05]	20.3 ± 1.2	22.5 ± 3.0	20.7 ± 0.8

Concentrations of the investigated analytes in the analysed wines according to sample set, vineyard origin, and harvest stage. Units are given in the respective table headings. The term “sample set” refers to the grouped Blaufränkisch/Mittelburgenland and Pinot Noir/Thermenregion materials used in the exploratory two-way ANOVA. Abbreviations: BF, Blaufränkisch; PN, Pinot Noir; LBG, Lutzmannsburg; HOR, Horitschon; GUN, Guntramsdorf; OBE, Oberwaltersdorf; EHD, early harvest date; SHD, standard harvest date; LHD, late harvest date. Exact harvest dates are given in Table 3. The corresponding basic must parameters, including °Brix, are presented in Table 4. Values are presented as mean ± SD of three independently harvested and vinified biological replicates unless otherwise indicated. Mean values and standard deviations are rounded for presentation; statistical analyses were performed using unrounded numerical concentration data. For calculated sum parameters, non-detected and non-quantifiable values were assigned a value of zero before summation on the original concentration scale; therefore, only quantifiable concentrations contributed positively to the respective sum. Symbols shown for sample set (S), harvest stage (H), and the S × H interaction are based on Benjamini–Hochberg-adjusted *p*-values from the exploratory two-way ANOVA of log10-transformed concentration data. Adjustment was performed separately across the eligible analytical endpoints for each model term. Bold *p*-values in square brackets indicate statistically detectable overall harvest-stage effects within the respective vineyard origin according to one-way ANOVA (*p* < 0.05). Different lowercase letters indicate statistically detectable differences between harvest stages within the same vineyard origin according to one-way ANOVA followed by Tukey’s HSD test (*p* < 0.05). These within-origin comparisons describe harvest-stage-related differences within each grape material and are not intended as direct comparisons between vineyard sites, wine-growing regions, or grape varieties. Symbol levels: * *p* < 0.05; ** *p* < 0.01; *** *p* < 0.001; n.s., not significant; n.a., not applicable; n.d., not detected. For *trans*-limonene oxide in OBE-EHD, the mean and standard deviation were calculated from the two quantifiable replicates; the third replicate was not detected.

**Table 10:** Concentrations of selected free C_13_-norisoprenoids in the investigated wines according to sample set, vineyard origin, and harvest stage.

Free C_13_-Norisoprenoids [µg/L]	Significance of Effects		Interaction	Sample Set	Vineyard Origin	Experimental Variants (Mean ± SD, *n* = 3)		
	Sample Set (S)	Harvest Stage (H)	S × H			EHD	SHD	LHD
Vitispirane	***	***	*	BF	LBG [*p* < **0.001**]	14.2 ^c^ ± 0.2	9.0 ^a^ ± 0.4	11.7 ^b^ ± 0.9
					HOR [*p* = **0.006**]	10.8 ^b^ ± 0.2	8.1 ^a^ ± 0.3	7.4 ^a^ ± 1.2
				PN	GUN [*p* < **0.001**]	3.2 ^c^ ± 0.2	1.6 ^b^ ± 0.1	1.3 ^a^ ± 0.1
					OBE [*p* < **0.001**]	2.4 ^c^ ± 0.0	1.9 ^b^ ± 0.0	1.5 ^a^ ± 0.0
TDN	n.a.	n.a.	n.a.	BF	LBG [*p* > 0.05]	0.4 ± 0.0	0.3 ± 0.1	0.3 ± 0.1
					HOR [*p* = **0.001**]	0.3 ^c^ ± 0.0	0.2 ^b^ ± 0.0	0.2 ^a^ ± 0.0
				PN	GUN	0.2 ± 0.0	0.1 ± 0.0	n.d.
					OBE [*p* < **0.001**]	0.1 ^b^ ± 0.0	0.1 ^b^ ± 0.0	0.1 ^a^ ± 0.0
β-ionone	***	***	n.s.	BF	LBG [*p* < **0.001**]	0.3 ^b^ ± 0.0	0.2 ^a^ ± 0.0	0.2 ^a^ ± 0.0
					HOR [*p* = **0.001**]	0.3 ^c^ ± 0.0	0.3 ^b^ ± 0.0	0.2 ^a^ ± 0.0
				PN	GUN [*p* = **0.006**]	0.5 ^b^ ± 0.1	0.3 ^a^ ± 0.0	0.4 ^a^ ± 0.0
					OBE [*p* = **0.003**]	0.5 ^b^ ± 0.0	0.3 ^a^ ± 0.0	0.3 ^a^ ± 0.1
β-damascenone	***	**	**	BF	LBG [*p* = **0.002**]	3.5 ^b^ ± 0.2	3.1 ^b^ ± 0.0	2.3 ^a^ ± 0.3
					HOR [*p* > 0.05]	2.9 ± 0.1	3.1 ± 0.2	2.6 ± 0.4
				PN	GUN [*p* < **0.001**]	2.6 ^a^ ± 0.2	6.0 ^c^ ± 0.4	4.8 ^b^ ± 0.2
					OBE [*p* = **0.001**]	5.0 ^a^ ± 0.2	7.1 ^b^ ± 0.4	6.3 ^b^ ± 0.4
∑ free C_13_-norisoprenoids	***	n.s.	**	BF	LBG [*p* < **0.001**]	18.4 ^c^ ± 0.3	12.6 ^a^ ± 0.4	14.5 ^b^ ± 1.1
					HOR [*p* = **0.005**]	14.4 ^b^ ± 0.2	11.6 ^a^ ± 0.6	10.4 ^a^ ± 1.3
				PN	GUN [*p* = **0.002**]	6.5 ^a^ ± 0.3	8.1 ^b^ ± 0.4	6.5 ^a^ ± 0.3
					OBE [*p* = **0.005**]	7.9 ^a^ ± 0.2	9.5 ^b^ ± 0.5	8.2 ^a^ ± 0.4

Concentrations of the investigated analytes in the analysed wines according to sample set, vineyard origin, and harvest stage. Units are given in the respective table headings. The term “sample set” refers to the grouped Blaufränkisch/Mittelburgenland and Pinot Noir/Thermenregion materials used in the exploratory two-way ANOVA. Abbreviations: BF, Blaufränkisch; PN, Pinot Noir; LBG, Lutzmannsburg; HOR, Horitschon; GUN, Guntramsdorf; OBE, Oberwaltersdorf; EHD, early harvest date; SHD, standard harvest date; LHD, late harvest date. Exact harvest dates are given in Table 3. The corresponding basic must parameters, including °Brix, are presented in Table 4. Values are presented as mean ± SD of three independently harvested and vinified biological replicates. Mean values and standard deviations are rounded for presentation; statistical analyses were performed using unrounded numerical concentration data. For calculated sum parameters, non-detected and non-quantifiable values were assigned a value of zero before summation on the original concentration scale; therefore, only quantifiable concentrations contributed positively to the respective sum. Symbols shown for sample set (S), harvest stage (H), and the S × H interaction are based on Benjamini–Hochberg-adjusted *p*-values from the exploratory two-way ANOVA of log10-transformed concentration data. Adjustment was performed separately across the eligible analytical endpoints for each model term. Bold *p*-values in square brackets indicate statistically detectable overall harvest-stage effects within the respective vineyard origin according to one-way ANOVA (*p* < 0.05). Different lowercase letters indicate statistically detectable differences between harvest stages within the same vineyard origin according to one-way ANOVA followed by Tukey’s HSD test (*p* < 0.05). These within-origin comparisons describe harvest-stage-related differences within each grape material and are not intended as direct comparisons between vineyard sites, wine-growing regions, or grape varieties. Symbol levels: * *p* < 0.05; ** *p* < 0.01; *** *p* < 0.001; n.s., not significant; n.a., not applicable; n.d., not detected.

## Data Availability

The original contributions presented in this study are included in the article. Further inquiries can be directed to the corresponding author. Aggregated concentration data and analytical performance characteristics supporting the findings of this study are presented within the article and its Appendix A. The underlying replicate-level data used for statistical analyses and multivariate evaluation are available from the corresponding author upon reasonable request.

## Appendix A

The analytical performance of the methods used in this study is documented in Appendix A. Table A1 summarises the available compound-specific method performance characteristics for the quantified aroma-related volatile compounds, including calibration range, linearity, limits of detection and quantification, repeatability or precision, and recovery where available. The data were compiled from the respective validated methods, published protocols, and supporting validation datasets for C_6_ alcohols, free monoterpenes, free C_13_-norisoprenoids, rotundone, and free volatile polyfunctional thiols.

Where compound-specific validation parameters were available, they are reported individually to support reproducibility and to document the suitability of the methods for the concentration ranges observed in the investigated wines. Parameters not available from the respective validation sources are indicated as not reported. For methods adopted or adapted from previously published protocols, the corresponding validation data and references are provided.

**Table A1:** Compound-specific analytical performance characteristics of the gas-chromatographic methods used for the quantification of selected aroma-related volatile compounds in the investigated wines.

Compound Class	Analyte	Unit	Analytical Approach	Quantifier/ Qualifier Ions or Transitions	Internal Standard	Calibration Range	Linearity	LOD	LOQ	Repeatability/Precision	Recovery	Source/Comment
C_6_ alcohols	1-Hexanol	mg/L	HS-SPME-GC-MS, partial SIDA	69/55	d13-hexanol	0.2–24.7	R^2^ = 0.999	n.r.	n.r.	2.8%	n.r.	In-house supplementary validation data; HS-SPME-GC-MS approach based on previously established aroma-compound methods
C_6_ alcohols	*cis*-3-Hexen-1-ol	mg/L	HS-SPME-GC-MS, partial SIDA	82/67, 55	d5-ethyl hexanoate	0.02–1.3	R^2^ = 0.999	n.r.	n.r.	11.0%	n.r.	In-house supplementary validation data; HS-SPME-GC-MS approach based on previously established aroma-compound methods
Free monoterpenes	*trans*-Linalool oxide	µg/L	HS-SPME-GC-SIM-MS	59/94, 111	3,4-dimethylanisole	0.5–100.6	R^2^ = 0.999	0.035	0.106	6.0%	n.r./matrix-dependent	Philipp et al. [34]; supporting validation data
Free monoterpenes	*cis*-Linalool oxide	µg/L	HS-SPME-GC-SIM-MS	59/94, 111	3,4-dimethylanisole	0.5–99.8	R^2^ = 0.999	0.173	0.525	5.8%	n.r./matrix-dependent	Philipp et al. [34]; supporting validation data
Free monoterpenes	*trans*-Limonene oxide	µg/L	HS-SPME-GC-SIM-MS	94/67, 109	3,4-dimethylanisole	0.6–117.7	R^2^ = 0.999	0.112	0.338	n.r.	n.r./matrix-dependent	Philipp et al. [34]; supporting validation data
Free monoterpenes	Linalool	µg/L	HS-SPME-GC-SIM-MS	71/93, 67	3,4-dimethylanisole	1.0–204	R^2^ = 0.999	0.028	0.086	7.0%	n.r./matrix-dependent	Philipp et al. [34]; supporting validation data
Free monoterpenes	Hotrienol	µg/L	HS-SPME-GC-SIM-MS	71/82, 67	3,4-dimethylanisole	0.5–109.7	R^2^ = 0.999	0.025	0.076	5.7%	n.r./matrix-dependent	Philipp et al. [34]; supporting validation data
Free monoterpenes	α-Terpineol	µg/L	HS-SPME-GC-SIM-MS	93/121, 136	3,4-dimethylanisole	0.7–133.0	R^2^ = 0.999	0.074	0.223	13.3%	n.r./matrix-dependent	Philipp et al. [34]; supporting validation data
Free monoterpenes	β-Terpineol	µg/L	HS-SPME-GC-SIM-MS	93/121, 136	3,4-dimethylanisole	0.1–18.3	R^2^ = 0.999	n.r.	n.r.	n.r.	n.r.	Philipp et al. [34]; supporting validation data; LOD/LOQ not available
Free monoterpenes	Nerol oxide	µg/L	HS-SPME-GC-SIM-MS	68/83	3,4-dimethylanisole	0.5–101.3	R^2^ = 0.999	0.014	0.043	4.1%	n.r./matrix-dependent	Philipp et al. [34]; supporting validation data
Free monoterpenes	Nerol	µg/L	HS-SPME-GC-SIM-MS	69/93, 95	3,4-dimethylanisole	1.0–202.5	R^2^ = 0.999	0.070	0.211	16.5%	n.r./matrix-dependent	Philipp et al. [34]; supporting validation data
Free monoterpenes	Citronellol	µg/L	HS-SPME-GC-SIM-MS	69/95, 93	3,4-dimethylanisole	1.0–201.5	R^2^ = 0.999	0.116	0.353	12.2%	n.r./matrix-dependent	Philipp et al. [34]; supporting validation data
Free monoterpenes	Geraniol	µg/L	HS-SPME-GC-SIM-MS	69/93, 123	3,4-dimethylanisole	1.0–203.7	R^2^ = 0.999	0.038	0.115	4.8%	n.r./matrix-dependent	Philipp et al. [34]; supporting validation data
Free C_13_-norisoprenoids	Vitispirane	µg/L	HS-SPME-GC-MS/MS	192→148, 192→168, 177→121	d5-vitispirane	0.01–52.25	R^2^ = 0.9994	0.00887	0.0269	3.437%	88.09%	In-house adaptation for free wine compounds based on Philipp et al. [2]; supporting validation data
Free C_13_-norisoprenoids	β-Ionone	µg/L	HS-SPME-GC-MS/MS	192→149, 177→162	d3-β-ionone	0.01–71.25	R^2^ = 0.9997	0.000591	0.00179	2.970%	91.25%	In-house adaptation for free wine compounds based on Philipp et al. [2]; supporting validation data
Free C_13_-norisoprenoids	TDN	µg/L	HS-SPME-GC-MS/MS	157→129, 157→172, 157→142	d5-TDN	0.01–50.25	R^2^ = 0.9980	0.000281	0.000851	7.543%	64.75%	In-house adaptation for free wine compounds based on Philipp et al. [2]; supporting validation data
Free C_13_-norisoprenoids	β-Damascenone	µg/L	HS-SPME-GC-MS/MS	190→175, 190→157, 190→117	d4-β-damascenone	0.01–48.08	R^2^ = 0.9988	0.00149	0.00452	4.922%	84.56%	In-house adaptation for free wine compounds based on Philipp et al. [2]; supporting validation data
Sesquiterpene	Rotundone	ng/L	SPE-SPME-SIDA-GC-MS/MS	218→163/218→161	d6-rotundone	0.5–100	R^2^ = 0.999	2	6	8.2%	97–108%	In-house supplementary validation data; method based on Philipp et al. [36]
Free volatile polyfunctional thiols	4-MSP	ng/L	SPE, derivatisation, LVI-PTV-GC-MS/MS	218→43, 100→43	4MSP-d10	1–500	R^2^ > 0.99	0.68	2.27	3.47–6.64%	87.8–126.7%	Method adapted from Sari et al. [37]; LOD and LOQ from in-house validation data
Free volatile polyfunctional thiols	3-SH	ng/L	SPE, derivatisation, LVI-PTV-GC-MS/MS	218→67, 173→61	3SH-d5	1–500	R^2^ > 0.99	0.93	3.10	1.74–3.03%	95.4–112.6%	Method adapted from Sari et al. [37]; LOD and LOQ from in-house validation data
Free volatile polyfunctional thiols	3-SHA	ng/L	SPE, derivatisation, LVI-PTV-GC-MS/MS	260→55, 200→59	3SHA-d5	1–500	R^2^ > 0.99	0.59	1.96	0.88–4.53%	96.7–106.8%	Method adapted from Sari et al. [37]; LOD and LOQ from in-house validation data

Abbreviations: n.r., not reported in the available validation source; SIDA, stable isotope dilution analysis; HS-SPME, headspace solid-phase microextraction; SPE, solid-phase extraction; LVI-PTV, large-volume injection–programmed temperature vaporisation; GC-MS/MS, gas chromatography–tandem mass spectrometry; 4-MSP, 4-methyl-4-sulfanylpentan-2-one; 3-SH, 3-sulfanylhexan-1-ol; 3-SHA, 3-sulfanylhexyl acetate; TDN, 1,1,6-trimethyl-1,2-dihydronaphthalene. The LOD and LOQ values are expressed in the units given in the “Unit” column for the respective analyte. The arrow (→) denotes the precursor-ion-to-product-ion transition used in multiple reaction monitoring (MRM). Repeatability or precision values are expressed as relative standard deviation (% RSD), where available. The notation “n.r./matrix-dependent” indicates that no general recovery value was reported because recovery or response behaviour was matrix-dependent. For C_6_ alcohols, compound-specific in-house supplementary validation data are reported where published validation data were not available for the exact analytes and wine matrix used in the present study. For free monoterpenes, method performance characteristics are based on Philipp et al. [34] and supporting validation data. For free C_13_-norisoprenoids, the method was applied to bottled wines for the determination of free compounds and was performed without acid hydrolysis; performance characteristics are based on an in-house adaptation of the approach described by Philipp et al. [2] and supporting validation data. Recovery values for free C_13_-norisoprenoids refer to the free-compound spiking experiment. For rotundone, performance characteristics refer to the SPE-SPME-SIDA-GC-MS/MS method applied to the present wine matrix. For free volatile polyfunctional thiols, the analytical method was adapted from Sari et al. [37]. The LOD and LOQ values reported here were obtained from subsequent in-house validation of the adapted method using signal-to-noise ratios of 3:1 and 10:1, respectively.
